# Interrupted Curtius Rearrangements of Quaternary Proline Derivatives: A Flow Route to Acyclic Ketones and Unsaturated Pyrrolidines

**DOI:** 10.1021/acs.joc.1c01133

**Published:** 2021-06-25

**Authors:** Marcus Baumann, Thomas S. Moody, Megan Smyth, Scott Wharry

**Affiliations:** † School of Chemistry, University College Dublin, Science Centre South, Belfield D04 N2E2, Ireland; ‡ Department of Technology, Almac Sciences, 20 Seagoe Industrial Estate, Craigavon BT63 5QD, United Kingdom; § Arran Chemical Company, Roscommon N37 DN24, Ireland

## Abstract

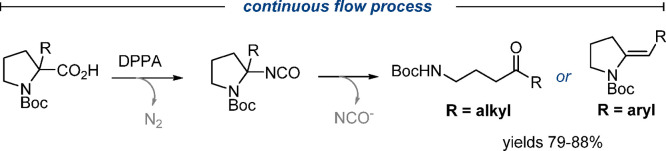

Conversion of *N*-Boc-protected quaternary proline derivatives under thermal Curtius rearrangement conditions was found to afford a series of ring-opened ketone and unsaturated pyrrolidine products instead of the expected carbamate species. The nature of the substituent on the quaternary carbon thereby governs the product outcome due to the stability of a postulated *N*-acyliminium species. A continuous flow process with in-line scavenging was furthermore developed to streamline this transformation and safely create products on a gram scale.

Since its first reports in the late 19th century, the Curtius rearrangement^1^ has established itself as one of the most versatile transformations to convert ubiquitous carboxylic acids into valuable amine derivatives.^2^ Acyl azide species (**1a**) are thereby the key intermediates in this process that release nitrogen upon rearranging into isocyanates that can be trapped with various nucleophiles (Scheme 1). Mechanistic studies support a concerted reaction pathway for thermal Curtius rearrangements, whereas photochemical alternatives proceed stepwise via nitrene intermediates.^3^ The popularity of the thermal Curtius rearrangement is evident from its regular application in natural product syntheses and drug development programs.^4^ Moreover, recent years have witnessed further developments by harnessing the salient features of continuous flow^5^ processing to yield modern variants^6^ that mitigate safety concerns due to the use of toxic azides, the release of nitrogen gas, and the potential for run-away phenomena related to the inherent exothermicity of this reaction sequence. Importantly, the exploitation of continuous flow processing has enabled several medicinal chemistry studies^7^ culminating in the safe execution of Curtius rearrangements on scales of >40 kg.^8^

**Scheme 1:**
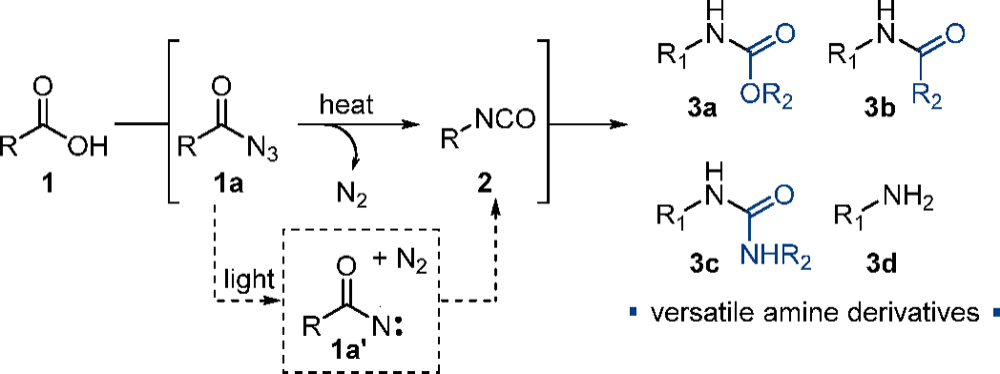
Overview of the Curtius Rearrangement

Despite these advances, essentially all studies on the Curtius rearrangement convert carboxylic acids into amines and amine derivatives such as carbamates, amides, and ureas (e.g., **3a**–**d**), thus overlooking opportunities for new directions. To address this shortcoming, this Note reports the realization of an interrupted Curtius rearrangement process rendering a set of new products such as γ-amino ketones and unsaturated pyrrolidines from Boc-protected proline derivatives.

Expanding on prior studies that integrated enzymatic impurity tagging strategies with flow-based Curtius rearrangement reactions,^9^ this work evaluated the use of Boc-protected proline species bearing substitution on the chiral carbon (e.g., **4**). Generation and subsequent trapping of the intermediate isocyanate **5** were thereby anticipated to afford a selection of versatile α-amino pyrrolidine species **6** as novel and potentially useful amine building blocks (Scheme 2).

**Scheme 2:**
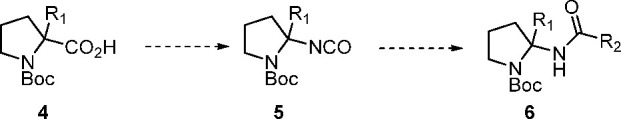
Intended Curtius Rearrangement Application for *N*-Boc Proline Derivatives

Commencing our study, the readily available proline derivative **4a** (R_1_ = Me)^10^ was subjected to standard Curtius rearrangement conditions using DPPA (diphenylphosporyl azide, **7**) as the azide source, triethylamine as the base, and benzyl alcohol as the trapping agent. Upon heating the reaction mixture in acetonitrile to reflux, the evolution of nitrogen gas was observed within minutes, indicating the onset of the anticipated rearrangement process. However, upon analyzing the crude reaction mixture by ^1^H NMR spectroscopy, not the anticipated Cbz product (R_2_ = OBn, **6a**), but a new ring-opened product was observed (entry 1, Table 1). This material was subsequently identified as ketone **9a** and confirmed by comparison to literature NMR data.^11^ As the evolution of nitrogen gas had indicated the generation of the anticipated isocyanate species, it was surmised that steric hindrance around the chiral center may have precluded nucleophilic attack by the modestly reactive benzyl alcohol. Reactions with other alcohols such as ethanol, methanol, and water (entries 2–4) resulted in the isolation of the same ketone product as the sole product in all three cases. Furthermore, alternative solvents such as toluene and dioxane did not alter the reaction outcome (entries 5 and 6). This outcome was reproduced even in the absence of an added alcohol nucleophile (entry 7). These results prompted consideration of an unprecedented reaction path that may be governed by the presence of the pyrrolidine ring as well as the quaternary center.

**Table 1:**
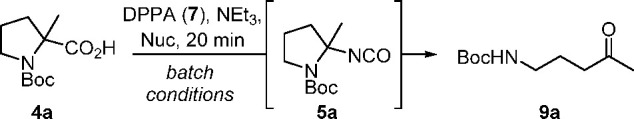
Formation of Ketone **9a** from *N*-Boc Proline Derivative **4a**

entry	solvent	nucleophile (Nuc)	yield (%)
1	MeCN	BnOH	85
2	MeCN	EtOH	81
3	MeCN	MeOH	77
4	MeCN	water	75
5	1,4-dioxane	BnOH	71
6	toluene	BnOH	84
7	MeCN	none	81

In view of the synthetic versatility of the 1,4-disubstitution pattern observed in this unexpected product and the desire to evaluate the generality of this process, a small selection of *N*-Boc-protected proline derivatives were subjected to these reaction conditions. Pleasingly, their syntheses were readily accomplished by lithiation of *N*-Boc proline methyl ester (**10**, 0.5 M in toluene, 1.0 equiv) with LiHMDS (1.0 M in THF, 1.1 equiv) as a base at −78 °C, followed by trapping the resulting carbanion with various electrophiles bearing alkyl and benzyl appendages (Scheme 3). Hydrolysis of the methyl ester (**11**) under alkaline conditions rendered the desired carboxylic acid building blocks **4** in high chemical yields.

**Scheme 3:**
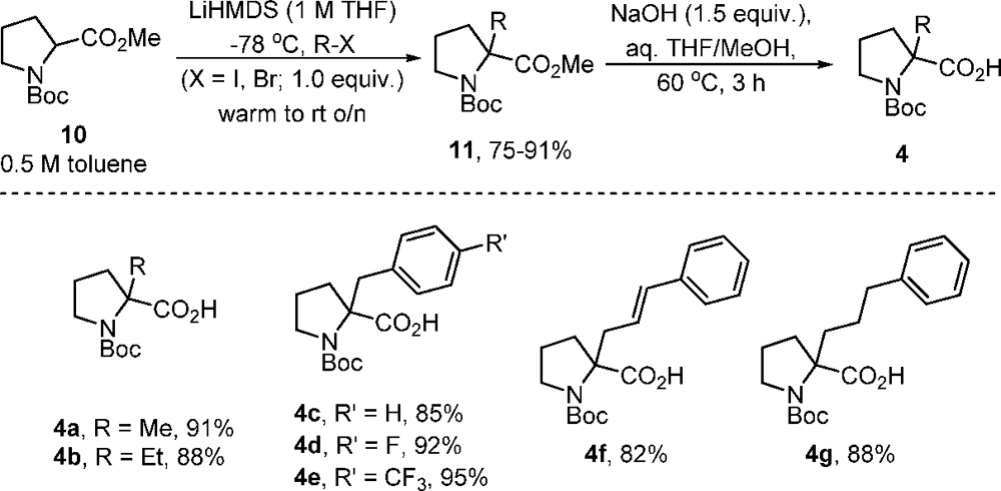
Batch Synthesis of Substrates **4** by Lithiation of *N*-Boc Proline Methyl Ester **10**

To streamline the following evaluation of the reaction scope, a flow reactor system was exploited to improve the process in view of safety, reproducibility, and scalability.^12^ In addition, toluene was preferred as a solvent in view of its higher boiling point and lower propensity to introduce water that may quench the acyl azide or isocyanate intermediate. Alcohols as nucleophiles were not added as they were found to have little effect on the reaction outcome (Table 1, entry 7). A simple flow process was devised and exploited in which streams of the substrate (**4a**–**g**, 1 M, toluene) in the presence of triethylamine as a base (1.0 equiv) and DPPA (**7**, 0.95 M, toluene, 0.95 equiv) were mixed in a T-piece before entering a heated flow coil (10 mL, 1/16 in id, PFA, 100 °C) with a residence time of 20 min (Scheme 4). The reaction mixture passed a back-pressure regulator (100 psi, Kinesis^13^) to ensure the steady release of nitrogen gas before collection in a receiving flask. A glass column containing a mixture of scavenger resins (Amberlyst A-21 and Amberlyst A-15;^14^ 2 g each per mmol of substrate **4a**–**g**) was optionally placed at the end of the reaction sequence to remove acidic and basic byproducts (NEt_3_, substrate, and diphenyl phosphonic acid).

**Scheme 4:**
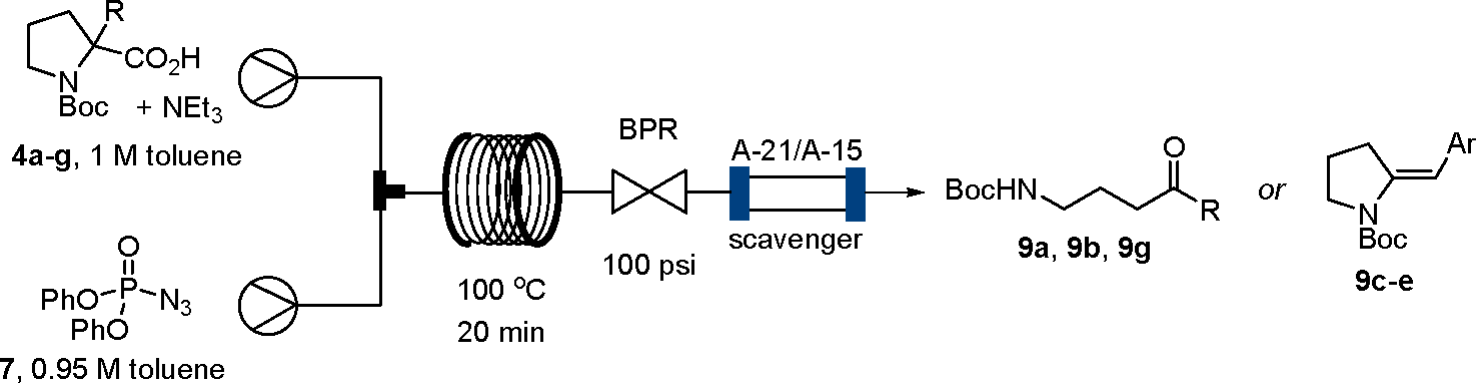
Flow Approach toward Evaluating the Reaction Scope of Interrupted Curtius Rearrangements

The resulting flow study confirmed that the initial substrate bearing a methyl substituent (**4a**) smoothly underwent the rearrangement process rendering methyl ketone **9a** in high chemical yield. Pleasingly, the same result was obtained when performing this process on a gram scale (Figure 1). Equally, introducing an ethyl group as a modified alkyl appendage was well tolerated and gave product **9b** in high yield. An interesting case was observed when subjecting cinnamyl analogue **4f** to the flow rearrangement protocol. As before, the formation of a gaseous byproduct (e.g., N_2_) was observed, and analysis of the crude material by ^1^H NMR indicated product formation in high yield; however, during purification by silica flash column chromatography, this material decomposed. It is surmised that the delicate skipped keto styryl moiety thereby tautomerized to a Michael acceptor, thus triggering decomposition by aldol or cycloaddition pathways. To support this assumption, cinnamyl ester **11f** was converted under transfer hydrogenation conditions^15^ into its saturated counterpart, and pleasingly, the corresponding acid **4g** furnished the anticipated ketone product (**9g**) in high yield and without any stability concerns. Furthermore, when subjecting unsubstituted *N*-Boc proline to the rearrangement process, decomposition of the labile aldehyde product (**9h**) was observed.

**Figure 1:**
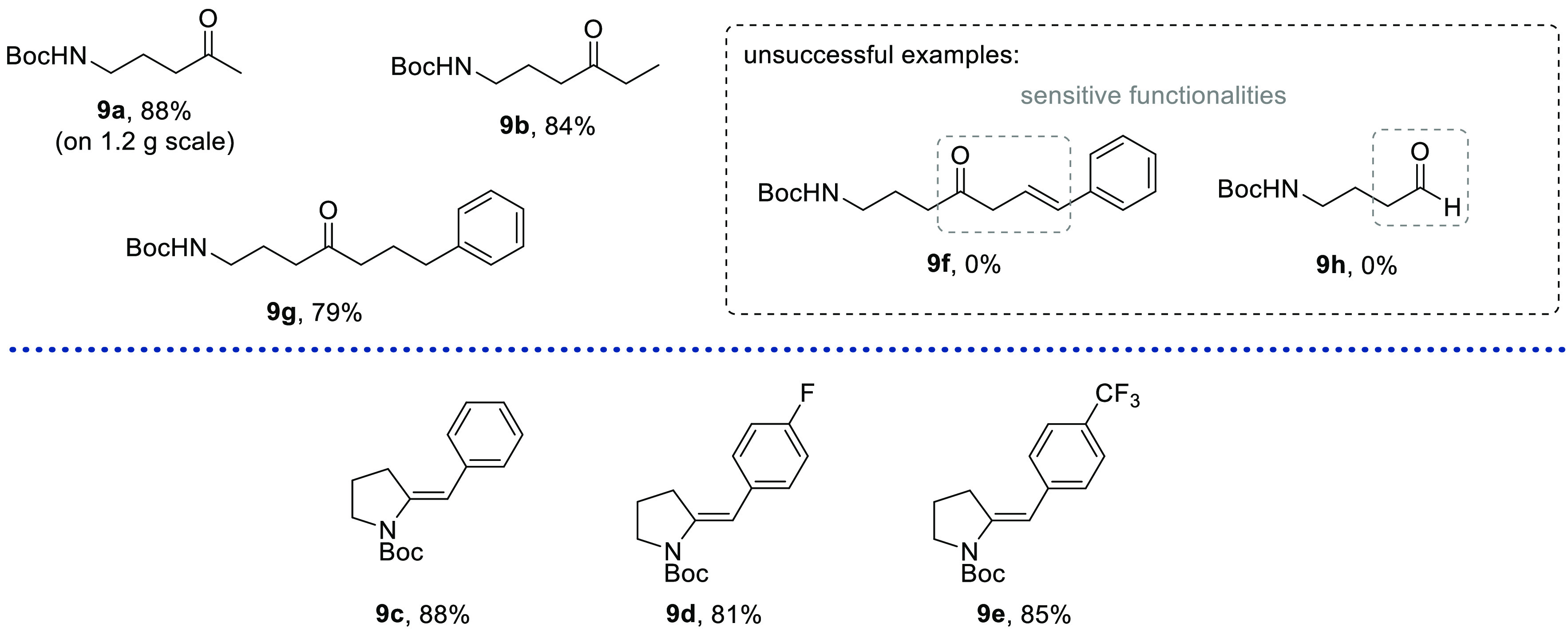
Reaction scope rendering acyclic products and unsaturated pyrrolidines.

In addition, several benzyl-substituted substrates (e.g., **4c**–**e**) were subjected to the flow protocol to verify if the analogous ring-opening process would take place. Formation of a gaseous byproduct was observed as before when employing the previous flow reaction conditions for this interrupted Curtius rearrangement process. However, when analyzing the purified reaction products (**9c**–**e**) by various NMR techniques, it was apparent that no ketone product was obtained. As these products were solids, single crystals were successfully grown for product **9e**, enabling X-ray diffraction experiments to unambiguously determine the correct connectivity of this product.^16^ This clearly indicated the presence of a partially unsaturated pyrrolidine ring bearing an exocyclic, *E*-configured alkene instead of a ring-opened product. Comparison of the NMR data of the other benzyl-derived products (**9c** and **9d**) indicated that the same product had formed as an exclusive *E*-isomer. Surprisingly relatively few mild and stereoselective methods for creating such unsaturated pyrrolidine scaffolds are reported in the literature. As these include examples that require transition metal catalysts and potentially render alternative alkene isomers or ring sizes,^17^ the methodology presented herein may serve as an attractive alternative.

To account for the observed reaction products, the following mechanism is proposed (Scheme 5). Activation of the carboxylic acid functionality with DPPA forms acyl azide **12**, which subsequently undergoes thermal rearrangement to an isocyanate (**5**) accompanied by the release of nitrogen gas. This quaternary isocyanate is assumed to be too hindered to undergo nucleophilic attack as anticipated in the regular Curtius rearrangement. In the absence of a strong base, it is proposed that a unimolecular process in which (iso)cyanate anion is expelled and cyclic acyliminium^18^ species **13** is obtained. This highly electrophilic acyliminium ion then reacts with adventitious water to give ketones (**9a**,**b**,**g**). In the case of a neighboring benzylic methylene group, the corresponding acyliminium appears to tautomerize rapidly by loss of the benzylic proton giving unsaturated pyrrolidine structures **9c**–**e** instead, which appear to be stable under the reaction conditions.^19^ It is believed that conjugation of the exocyclic alkene into the benzene ring for products **9c**–**e** imparts higher stability toward attack by nucleophiles such as water and thus accounts for the observed reaction outcome.

**Scheme 5:**
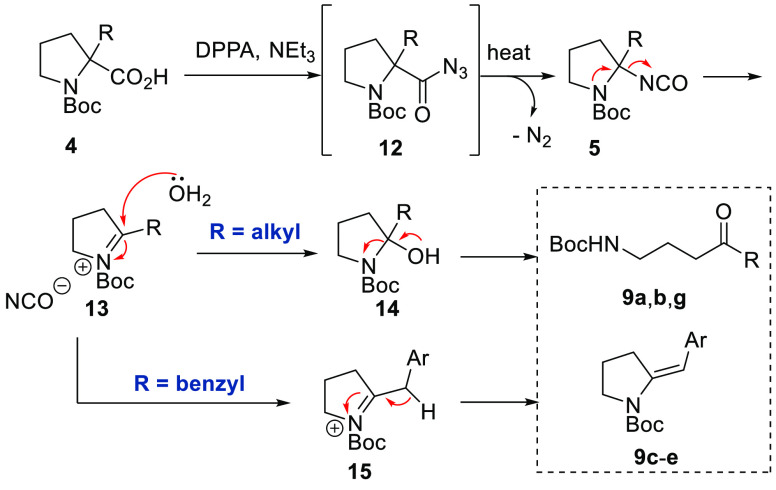
Proposed Reaction Mechanism Accounting for Bifurcated Pathway

In conclusion, a novel reaction pathway for quaternary *N*-Boc proline species under thermal Curtius rearrangement conditions is reported. Fragmentation of the intermediate isocyanate species thereby renders a proposed *N*-acyliminium species, which facilitates ring-opening by adventitious water to give γ-amino ketone products in the case of small aliphatic substituents, whereas benzylic appendages render unsaturated pyrrolidines via tautomerization of this proposed *N*-acyliminium intermediate. A continuous flow protocol was successfully established to enable the safe and scaled exploration of this transformation. In view of its simplicity, high yields, and the value of the generated reaction products, this interrupted Curtius rearrangement method may find future synthetic applications in both batch and flow mode.

## Experimental Section

### Materials and Methods

Unless otherwise stated, all solvents were purchased from Fisher Scientific and used without further purification. Substrates and reagents were purchased from Fluorochem or Sigma-Aldrich and used as received.

The heating of reaction mixtures was achieved by using DrySyn metal heating blocks available from Asynt.

^1^H NMR spectra were recorded on 300, 400, and 500 MHz instruments and are reported relative to the residual solvent: CHCl_3_ (δ 7.26 ppm). ^13^C{^1^H} NMR spectra were recorded on the same instruments (100 and 125 MHz) and are reported relative to CHCl_3_ (δ 77.16 ppm). ^19^F NMR were recorded at 282 and 376 MHz. Data for ^1^H NMR are reported as follows: chemical shift (δ/ ppm) (integration, multiplicity, coupling constant (Hz)). Multiplicities are reported as follows: s = singlet, d = doublet, t = triplet, q = quartet, p = pentet, m = multiplet, br s = broad singlet, app = apparent. Data for ^13^C{^1^H} NMR are reported in terms of chemical shift (δ/ppm) and multiplicity (C, CH, CH_2_, or CH_3_). COSY and HSQC experiments were used in the structural assignment.

IR spectra were obtained by use of a Bruker Platinum spectrometer (neat, ATR sampling) with the intensities of the characteristic signals being reported as weak (w, <20% of the tallest signal), medium (m, 21–70% of the tallest signal), or strong (s, >71% of the tallest signal).

High-resolution mass spectrometry was performed using the indicated techniques on a micromass LCT orthogonal time-of-flight mass spectrometer with leucine-enkephalin (Tyr-Gly-Phe-Leu) as an internal lock mass. GC–MS was performed on a Waters GCT Premier Agilent 7898 system (column Macherey–Nagel; Optima 5 MS, length 15 m, diameter 0.25 mm).

Melting points were recorded on a Stuart SMP10 melting point apparatus and are uncorrected.

Continuous flow experiments were performed on a Vaportec E-series system in combination with Omnifit glass columns (6.6 mm id, 150 mm length) filled with scavenging resins (A-15/A-21).

### Synthetic Procedures and Spectroscopic Data

#### Synthesis of Substituted *N*-Boc Proline Derivatives **11b**–**h**

For a reaction on 2 mmol scale, to a solution of the *N*-Boc proline methyl ester (**10**, 1.0 equiv, 2.0 mmol) in toluene (0.5 M, held at −78 °C) was added a solution of LiHMDS (1 M, THF, 1.1 equiv, 2.2 mmol) dropwise. The resulting mixture was allowed to warm to 0 °C within 20 min before cooling to −78 °C. A solution of alkyl bromide or iodide electrophile (0.5 M, toluene, 1.0 equiv, 2.0 mmol) was subsequently added dropwise, and the reaction mixture continued stirring for 12 h, eventually warming to rt. The reaction mixture was quenched by the addition of NH_4_Cl solution (sat. aq) and extracted (DCM/water). The combined organic layers were dried over anhydrous Na_2_SO_4_, filtered, and evaporated under reduced pressure to give the crude product. Purification was achieved via silica gel chromatography using a cyclohexane/ethyl acetate (75:15) eluent system.

#### 1-(*tert*-Butyl) 2-Methyl 2-ethylpyrrolidine-1,2-dicarboxylate (**11b**).^20^

Yield: 640 mg (3.6 mmol, 83%). Appearance: clear oil. Mixtures of Boc-rotamers were observed by NMR spectroscopy; please see copies of ^1^H and ^13^C NMR spectra in Supporting Information. ^1^H NMR (400 MHz, CDCl_3_): δ 3.75–3.69 (m), 3.66 (s), 3.63–3.55 (m), 3.42–3.31 (m), 2.37–2.25 (m), 2.20–2.10 (m), 2.06–1.94 (m), 1.92–1.74 (m), 1.42 (s), 1.37 (s), 0.88–0.80 (m). ^13^C{^1^H} NMR (101 MHz, CDCl_3_): δ 175.6, 175.4, 154.0, 153.8, 79.8, 79.3, 68.3, 67.8, 52.0, 51.9, 48.7, 48.6, 36.9, 35.6, 28.4, 28.3, 27.8, 26.6, 23.2, 22.7, 7.9. IR (neat): ν/cm^–1^ 2974 (m), 2879 (w), 1741 (m), 1693 (s), 1455 (m), 1386 (s), 1234 (m), 1157 (s), 1130 (s), 1075 (m), 1002 (m), 772 (m). HRMS (TOF-ESI+): calcd for C_13_H_23_NO_4_Na, 280.1519; found, 280.1522 (M + Na^+^).

#### 1-(*tert*-Butyl) 2-Methyl 2-benzylpyrrolidine-1,2-dicarboxylate (**11c**)

Yield: 870 mg (2.7 mmol, 91%). Appearance: clear oil. Mixtures of Boc-rotamers were observed by NMR spectroscopy; please see copies of ^1^H and ^13^C NMR spectra in Supporting Information. ^1^H NMR (500 MHz, CDCl_3_): δ 7.30–7.21 (m), 7.14 (td, *J* = 6.8, 5.9, 3.2 Hz), 3.77 (d, *J* = 14.0 Hz), 3.75 (s), 3.57 (d, *J* = 13.8 Hz), 3.48 (dt, *J* = 10.5, 7.5 Hz), 3.39 (dt, *J* = 10.4, 7.2 Hz), 3.05 (d, *J* = 13.8 Hz), 3.04 (d, *J* = 13.8 Hz), 2.99 (ddd, *J* = 10.5, 7.6, 5.3 Hz), 2.88 (ddd, *J* = 10.4, 7.6, 5.6 Hz), 2.12–1.97 (m), 1.58 (ddt, *J* = 18.2, 7.4, 5.2 Hz), 1.51 (s), 1.49 (s), 1.00–0.83 (m). ^13^C{^1^H} NMR (126 MHz, CDCl_3_): δ 175.3, 175.1, 154.1, 153.5, 137.3, 136.9, 130.8, 130.7, 128.2, 128.0, 126.6, 126.4, 80.3, 79.6, 68.3, 68.0, 52.3, 48.2, 39.7, 38.5, 36.6, 35.4, 28.5, 28.4, 22.8, 22.2. IR (neat): ν/cm^–1^ 2975 (m), 2877 (w), 1741 (m), 1694 (s), 1454 (m), 1389 (s), 1366 (m), 1251 (m), 1168 (s), 1119 (m), 1020 (m), 704 (m). HRMS (TOF-ESI+): calcd for C_18_H_25_NO_4_Na, 342.1676; found, 342.1679 (M + Na^+^).

#### 1-(*tert*-Butyl) 2-Methyl 2-(4-fluorobenzyl)pyrrolidine-1,2-dicarboxylate (**11d**)

Yield: 750 mg (2.3 mmol, 75%). Appearance: clear oil. Mixtures of Boc-rotamers were observed by NMR spectroscopy; please see copies of ^1^H and ^13^C NMR spectra in Supporting Information. ^1^H NMR (500 MHz, CDCl_3_): δ 7.11–7.06 (m), 6.98–6.92 (m), 3.74 (s), 3.73 (s), 3.54–3.45 (m), 3.40 (dt, *J* = 10.6, 7.2 Hz), 3.03 (d, *J* = 14.2 Hz), 3.01 (d, *J* = 14.2 Hz), 3.00–2.96 (m), 2.89 (ddd, *J* = 10.4, 7.4, 5.7 Hz), 2.09–1.98 (m), 1.65–1.55 (m), 1.49 (s), 1.47 (s), 1.05–0.87 (m). ^13^C{^1^H} NMR (126 MHz, CDCl_3_): δ 175.1, 175.0, 161.9 (CF, d, *J* = 246 Hz), 161.8 (CF, d, *J* = 246 Hz), 154.2, 153.5, 132.9 (d, *J* = 4 Hz), 132.6 (d, *J* = 4 Hz), 132.1 (d, *J* = 8 Hz), 132.0 (d, *J* = 8 Hz), 115.1 (d, *J* = 21 Hz), 114.8 (d, *J* = 21 Hz), 80.4, 79.7, 68.3, 67.9, 52.3, 52.3, 48.3, 48.2, 38.9, 37.7, 36.5, 35.3, 28.4, 28.4, 22.8, 22.2. IR (neat): ν/cm^–1^ 2976 (m), 2877 (w), 1741 (s), 1693 (s), 1510 (s), 1388 (s), 1251 (m), 1221 (m), 1160 (s), 1131 (m), 1016 (m), 844 (m), 772 (m). HRMS (TOF-ESI+): calcd for C_18_H_24_FNO_4_Na, 360.1582; found, 360.1585 (M + Na^+^).

#### 1-(*tert*-Butyl) 2-Methyl 2-(4-(trifluoromethyl)benzyl)pyrrolidine-1,2-dicarboxylate (**11e**)

Yield: 920 mg (2.4 mmol, 80%). Appearance: white solid. Melting range: 94–96 °C. Mixtures of Boc-rotamers were observed by NMR spectroscopy; please see copies of ^1^H and ^13^C NMR spectra in Supporting Information. ^1^H NMR (400 MHz, CDCl_3_): δ 7.48 (dd, *J* = 8.2, 4.3 Hz), 7.22 (d, *J* = 7.9 Hz), 3.78 (d, *J* = 13.8 Hz), 3.71 (s), 3.70 (s), 3.56 (d, *J* = 13.8 Hz), 3.47 (dt, *J* = 10.6, 7.3 Hz), 3.37 (dt, *J* = 10.5, 7.1 Hz), 3.08 (d, *J* = 13.8 Hz), 3.07 (d, *J* = 13.8 Hz), 2.95 (ddd, *J* = 10.5, 7.6, 5.5 Hz), 2.86 (ddd, *J* = 10.4, 7.4, 5.8 Hz), 2.10–1.94 (m), 1.64–1.54 (m), 1.45 (s), 1.43 (s), 1.01–0.86 (m). ^13^C{^1^H} NMR (101 MHz, CDCl_3_): δ 174.8, 174.7, 154.2, 153.4, 141.5, 141.2, 129.0 (q, *J* = 32 Hz), 128.8 (q, *J* = 32 Hz), 125.0 (q, *J* = 4 Hz), 124.8 (q, *J* = 4 Hz), 124.3 (CF_3_, q, *J* = 273 Hz), 124.2 (CF_3_, q, *J* = 273 Hz), 80.5, 79.8, 68.1, 67.8, 52.3, 48.2, 39.7, 38.4, 36.6, 35.3, 28.3, 28.3, 22.7, 22.2.IR (neat): ν/cm^–1^ 2977 (w), 2880 (w), 1742 (m), 1694 (s), 1389 (s), 1324 (s), 1251 (m), 1163 (s), 1123 (s), 1111 (s), 1067 (s), 1019 (m), 851 (m). HRMS (TOF-ESI+): calcd for C_19_H_24_NF_3_O_4_Na, 410.1550; found, 410.1551 (M + Na^+^).

#### 1-(*tert*-Butyl) 2-Methyl 2-cinnamylpyrrolidine-1,2-dicarboxylate (**11f**)

Yield: 550 mg (1.6 mmol, 81%). Appearance: clear oil. Mixtures of Boc-rotamers were observed by NMR spectroscopy; please see copies of ^1^H and ^13^C NMR spectra in Supporting Information. ^1^H NMR (300 MHz, CDCl_3_): δ 7.40–7.18 (m), 6.48 (app d, *J* = 15.7 Hz), 6.27–6.08 (m), 3.76 (s, 3H), 3.73–3.52 (m), 3.48–3.32 (m), 3.26 (dd, *J* = 14.1, 6.9 Hz), 3.08 (dd, *J* = 14.3, 6.3 Hz), 2.81 (d, *J* = 9.1 Hz), 2.76 (d, *J* = 8.6 Hz), 2.23–2.05 (m), 1.96–1.73 (m), 1.48 (s), 1.46 (s). ^13^C{^1^H} NMR (126 MHz, CDCl_3_): δ 175.1, 174.9, 154.0, 153.6, 137.6, 137.3, 133.9, 133.7, 128.6, 128.5, 127.3, 127.1, 126.3, 126.1, 125.3, 124.9, 80.2, 79.6, 67.9, 67.3, 52.2, 52.2, 48.5, 48.5, 38.9, 37.6, 37.1, 35.9, 28.4, 28.4, 23.2, 22.7. IR (neat): ν/cm^–1^ 2975 (m), 2876 (w), 1741 (s), 1697 (s), 1390 (s), 1249 (m), 1164 (m), 1135 (m), 748 (w), 695 (w). HRMS (TOF-ESI+): calcd for C_20_H_27_NO_4_Na, 368.1832; found, 368.1835 (M + Na^+^).

#### 1-(*tert*-Butyl) 2-Methyl 2-(3-phenylpropyl)pyrrolidine-1,2-dicarboxylate (**11g**)

To a solution of ester **11f** (1 mmol, 1.0 equiv) in EtOAc (10 mL, 0.1 M) were added ammonium formate (5 mmol, 5.0 equiv) and Pd/C (10%, ca. 100 mg). This solution was then heated under reflux for 5 h when TLC indicated full conversion of the substrate. After cooling to room temperature, the mixture was filtered through a pad of Celite, washed with EtOAc (10 mL), and concentrated *in vacuo* to give the saturated product **11g** (quant.).

Yield: 344 mg (1.0 mmol, 99%). Appearance: clear oil. Mixtures of Boc-rotamers were observed by NMR spectroscopy; please see copies of ^1^H and ^13^C NMR spectra in Supporting Information. ^1^H NMR (500 MHz, CDCl_3_): δ 7.32–7.24 (m), 7.19 (app dd, *J* = 8.2, 4.4 Hz), 3.75–3.71 (m), 3.70 (s), 3.69 (s), 3.64–3.56 (m), 3.42–3.30 (m), 2.74–2.56 (m), 2.38 (ddd, *J* = 14.0, 12.2, 4.8 Hz), 2.20 (td, *J* = 13.3, 4.4 Hz), 2.13–1.94 (m), 1.94–1.84 (m), 1.79 (tq, *J* = 12.6, 6.5 Hz), 1.71–1.60 (m), 1.60–1.51 (m), 1.45 (s), 1.33 (s). ^13^C{^1^H} NMR (126 MHz, CDCl_3_): δ 175.5, 175.3, 154.0, 153.8, 142.6, 142.1, 128.5, 128.4, 128.3, 128.3, 125.8, 125.7, 79.8, 79.4, 67.9, 67.4, 52.1, 52.0, 48.6, 48.5, 37.5, 36.2, 36.1, 34.7, 33.9, 28.4, 28.2, 25.9, 25.4, 23.2, 22.8. IR (neat): ν/cm^–1^ 2974 (m), 2870 (w), 1741 (m), 1697 (s), 1454 (w), 1391 (s), 1366 (m), 1235 (m), 1163 (m), 1132 (m), 700 (w). HRMS (TOF-ESI+): calcd for C_20_H_29_NO_4_Na, 370.1989; found, 370.1994 (M + Na^+^).

#### Synthesis of Acid Products **4a**–**g**

For a reaction on 2 mmol scale, to a solution of ester intermediate **11** (2 mmol, 1.0 equiv, 2 M, THF/MeOH, 50:50 vol) was added a solution of NaOH (1 M aqueous, 4 mL, 2.0 equiv). The resulting mixture was heated to reflux until TLC indicated full consumption of the substrate (ca. 3 h). The resulting solution was partitioned between EtOAc and water. The aqueous layer was subsequently acidified by the addition of 1 M HCl (ca. 5 mL) and extracted with DCM, giving the target acid products after drying of the organic layer over anhydrous Na_2_SO_4_, filtration, and final evaporation under reduced pressure.

#### 1-(*tert*-Butoxycarbonyl)-2-methylpyrrolidine-2-carboxylic Acid (**4a**).^21^

Yield: 2.5 g (10.9 mmol, 91%). Appearance: colorless crystalline solid. Melting range: 129–131 °C (CHCl_3_). Mixtures of Boc-rotamers were observed by NMR spectroscopy; please see copies of ^1^H and ^13^C NMR spectra in Supporting Information. ^1^H NMR (500 MHz, CDCl_3_): δ 11.67 (br s), 3.60–3.39 (m), 2.38–2.30 (m), 2.26–2.22 (m), 1.92 (app tt, *J* = 10.2, 5.4 Hz), 1.86–1.79 (m), 1.55 (s), 1.48 (s), 1.42 (s), 1.38 (s). ^13^C{^1^H} NMR (126 MHz, CDCl_3_): δ 180.7, 179.0, 154.9, 153.7, 80.4, 80.4, 65.6, 64.8, 48.2, 47.8, 40.3, 39.0, 28.4, 28.3, 23.0, 22.8, 22.7, 22.1. IR (neat): ν/cm^–1^ 3200–2800 (br), 2977 (m), 2884 (m), 1731 (s), 1619 (s), 1446 (m), 1417 (s), 1392 (s), 1366 (s), 1248 (m), 1159 (s), 1138 (s), 1080 (m), 892 (m), 852 (m), 772 (m), 583 (m). HRMS (TOF-ESI+): calcd for C_11_H_20_NO_4_, 230.1387; found, 230.1388 (M + H^+^).

#### 1-(*tert*-Butoxycarbonyl)-2-ethylpyrrolidine-2-carboxylic Acid (**4b**)

Yield: 0.5 g (2.1 mmol, 88%). Appearance: clear oil. Mixtures of Boc-rotamers were observed by NMR spectroscopy; please see copies of ^1^H and ^13^C NMR spectra in Supporting Information. ^1^H NMR (400 MHz, CDCl_3_): δ 3.73–3.67 (m), 3.54–3.46 (m), 3.37 (dt, *J* = 10.7, 7.5 Hz), 3.29 (dt, *J* = 10.3, 8.0 Hz), 2.65–2.48 (m), 2.21–2.03 (m), 2.03–1.85 (m), 1.81–1.71 (m), 1.45 (s), 1.38 (s), 0.84 (app t, *J* = 7.5 Hz). ^13^C{^1^H} NMR (101 MHz, CDCl_3_): δ 180.9, 175.4, 156.9, 153.9, 81.8, 80.2, 70.8, 67.7, 49.4, 48.6, 36.9, 34.5, 28.3, 28.3, 27.4, 27.1, 22.7, 8.1, 7.7. IR (neat): ν/cm^–1^ 3400–2400 (br), 2974 (m), 2880 (w), 1741 (m), 1698 (s), 1391 (s), 1367 (s), 1161 (s), 931 (m), 858 (m), 773 (m). HRMS (TOF-ESI+): calcd for C_12_H_21_NO_4_Na, 244.1543; found, 244.1550 (M + Na^+^).

#### 2-Benzyl-1-(*tert*-butoxycarbonyl)pyrrolidine-2-carboxylic Acid (**4c**)

Yield: 0.7 g (2.3 mmol, 85%). Appearance: clear oil. Mixtures of Boc-rotamers were observed by NMR spectroscopy; please see copies of ^1^H and ^13^C NMR spectra in Supporting Information. ^1^H NMR (500 MHz, CDCl_3_): δ 7.31–7.22 (m,), 7.17–7.13 (m), 3.67 (d, *J* = 13.9 Hz), 3.61 (d, *J* = 13.8 Hz), 3.51 (dt, *J* = 10.5, 7.3 Hz), 3.40 (ddd, *J* = 10.7, 7.5, 5.0 Hz), 3.10 (d, *J* = 13.7 Hz), 3.03 (d, *J* = 14.0 Hz), 3.01–2.96 (m), 2.93 (dt, *J* = 10.7, 7.5 Hz), 2.36 (ddd, *J* = 13.0, 7.2, 5.6 Hz), 2.21–2.09 (m), 2.01 (ddd, *J* = 13.0, 8.3, 7.1 Hz), 1.68–1.59 (m), 1.54 (s), 1.52 (s), 1.19 (tdd, *J* = 12.4, 7.0, 5.3 Hz), 0.93 (dp, *J* = 12.3, 7.5 Hz). ^13^C{^1^H} NMR (126 MHz, CDCl_3_): δ 180.6, 177.1, 156.1, 153.6, 136.7, 136.2, 130.7, 130.6, 128.3, 128.2, 126.8, 126.7, 81.3, 80.8, 69.8, 68.0, 49.0, 48.3, 39.4, 38.6, 36.7, 34.7, 28.5, 28.4, 22.4, 22.2. IR (neat): ν/cm^–1^ 3300–2700 (br), 2976 (m), 2878 (m), 1737 (m), 1697 (s), 1392 (s), 1368 (m), 1167 (s), 1139 (m), 994 (m), 703 (m). HRMS (TOF-ESI+): calcd for C_17_H_24_NO_4_, 306.1700; found, 306.1705 (M + H^+^).

#### 1-(*tert*-Butoxycarbonyl)-2-(4-fluorobenzyl)pyrrolidine-2-carboxylic Acid (**4d**)

Yield: 0.5 g (1.5 mmol, 92%). Appearance: waxy white solid. Mixtures of Boc-rotamers were observed by NMR spectroscopy; please see copies of ^1^H and ^13^C NMR spectra in Supporting Information. ^1^H NMR (500 MHz, CDCl_3_): δ 7.16–7.09 (m), 7.01–6.94 (m), 3.67 (d, *J* = 13.9 Hz), 3.59–3.50 (m), 3.43 (ddd, *J* = 10.6, 7.5, 5.2 Hz), 3.08 (d, *J* = 14.0 Hz), 3.05–2.98 (m), 2.94 (dt, *J* = 10.7, 7.3 Hz), 2.30 (ddd, *J* = 13.2, 7.2, 5.9 Hz), 2.18 (dt, *J* = 13.5, 7.7 Hz), 2.11–1.95 (m), 1.73–1.63 (m), 1.53 (s), 1.51 (s), 1.29–1.18 (m), 0.99 (dp, *J* = 12.1, 7.5 Hz). ^13^C{^1^H} NMR (126 MHz, CDCl_3_): δ 180.8, 178.0, 161.9 (2xd, *J* = 247 Hz), 155.6, 153.6, 132.3 (2x), 132.0 (m), 115.2 (d, *J* = 21 Hz), 115.1 (d, *J* = 21 Hz), 81.0, 81.0, 69.2, 67.9, 48.8, 48.3, 38.6, 37.6, 36.7, 34.9, 28.5, 28.4, 22.5, 22.3. IR (neat): ν/cm^–1^ 3300–2600 (br), 2977 (m), 2880 (w), 1737 (m), 1696 (s), 1510 (s), 1392 (s), 1368 (s), 1223 (s), 1160 (s), 1140 (m), 996 (m), 840 (m), 771 (m). HRMS (TOF-ESI+): calcd for C_17_H_23_FNO_4_, 324.1611; found, 324.1618 (M + H^+^).

#### 1-(*tert*-Butoxycarbonyl)-2-(4-(trifluoromethyl)benzyl)pyrrolidine-2-carboxylic Acid (**4e**)

Yield: 0.6 g (1.6 mmol, 95%). Appearance: waxy solid. Mixtures of Boc-rotamers were observed by NMR spectroscopy; please see copies of ^1^H and ^13^C NMR spectra in Supporting Information. ^1^H NMR (400 MHz, CDCl_3_): δ 7.56–7.52 (m), 7.28–7.24 (m), 3.69 (d, *J* = 13.7 Hz), 3.62 (d, *J* = 13.9 Hz), 3.53 (dt, *J* = 10.5, 7.3 Hz), 3.46–3.39 (m), 3.18 (d, *J* = 13.6 Hz), 3.08 (d, *J* = 13.9 Hz), 3.03–2.91 (m), 2.37 (dt, *J* = 12.7, 6.6 Hz), 2.24–2.15 (m), 2.08–2.02 (m), 1.98–1.88 (m), 1.73–1.58 (m), 1.52 (s), 1.48 (s), 1.31–1.21 (m), 1.00 (dt, *J* = 12.5, 7.3 Hz). ^13^C{^1^H} NMR (101 MHz, CDCl_3_): δ 179.9, 176.3, 156.1, 153.5, 140.9, 140.3, 131.0, 130.8, 125.2, 125.1, 125.1, 81.6, 81.1, 49.0, 48.3, 39.4, 38.5, 36.7, 34.7, 28.4, 28.4, 22.4, 22.3, 20.6; some resonances were not observed clearly. IR (neat): ν/cm^–1^ 3400–2700 (br), 2977 (m), 2882 (w), 1738 (m), 1696 (s), 1392 (s), 1324 (s), 1163 (s), 1124 (s), 1112 (s), 1068 (s), 851 (m). HRMS (TOF-ESI+): calcd for C_18_H_22_F_3_NO_4_Na, 396.1393; found, 396.1393 (M + Na^+^).

#### 1-(*tert*-Butoxycarbonyl)-2-cinnamylpyrrolidine-2-carboxylic Acid (**4f**)

Yield: 0.6 g (1.8 mmol, 82%). Appearance: clear oil. Mixtures of Boc-rotamers were observed by NMR spectroscopy; please see copies of ^1^H and ^13^C NMR spectra in Supporting Information. ^1^H NMR (300 MHz, CDCl_3_): δ 7.44–7.21 (m), 6.53 (d, *J* = 15.7 Hz), 6.04 (dt, *J* = 15.5, 7.5 Hz), 3.59–3.48 (m), 3.38–3.28 (m), 3.09 (dd, *J* = 13.7, 7.9 Hz), 2.88 (dd, *J* = 13.9, 7.1 Hz), 2.80–2.67 (m), 2.27–2.17 (m), 2.05–1.92 (m), 1.85–1.74 (m), 1.51 (app s). ^13^C{^1^H} NMR (126 MHz, CDCl_3_): δ 180.3, 175.9, 156.5, 153.7, 137.2, 137.2, 134.8, 134.1, 128.6, 127.5, 127.4, 126.2, 126.2, 124.7, 123.3, 81.7, 80.7, 69.6, 67.3, 49.3, 48.5, 38.5, 37.3, 37.2, 34.9, 28.4, 28.4, 22.7, 22.7. IR (neat): ν/cm^–1^ 3300–2700 (br), 2975 (m), 2879 (w), 1733 (m), 1695 (s), 1390 (s), 1367 (s), 1247 (m), 1164 (s), 998 (m), 969 (m), 734 (s), 694 (s). HRMS (TOF-ESI+): calcd for C_19_H_25_NO_4_Na, 354.1676; found, 354.1677 (M + Na^+^).

#### 1-(*tert*-Butoxycarbonyl)-2-(3-phenylpropyl)pyrrolidine-2-carboxylic Acid (**4g**)

Yield: 0.4 g (1.2 mmol, 88%). Appearance: clear oil. Mixtures of Boc-rotamers were observed by NMR spectroscopy; please see copies of ^1^H and ^13^C NMR spectra in Supporting Information. ^1^H NMR (400 MHz, CDCl_3_): δ 7.29–7.23 (m), 7.20–7.12 (m), 3.75–3.65 (m), 3.54–3.45 (m), 3.37 (dt, *J* = 10.9, 7.4 Hz), 3.32–3.22 (m), 2.74–2.54 (m), 2.25–1.98 (m), 1.94–1.72 (m), 1.69–1.50 (m,), 1.47 (s), 1.31 (s). ^13^C{^1^H} NMR (101 MHz, CDCl_3_): δ 180.9, 175.7, 156.7, 153.8, 142.0, 141.9, 128.4, 128.3, 128.3, 125.9, 125.8, 81.8, 80.3, 70.0, 67.3, 49.2, 48.5, 37.5, 36.0, 35.8, 35.2, 34.3, 33.9, 28.4, 28.2, 26.0, 25.3, 22.7, 22.7. IR (neat): ν/cm^–1^ 3250–2600 (br), 2973 (m), 2932 (m), 2873 (w), 1738 (m), 1694 (s), 1453 (m), 1389 (s), 1366 (s), 1244 (m), 1162 (s), 1136 (s), 856 (m), 749 (m), 698 (s). HRMS (TOF-ESI+): calcd for C_19_H_28_NO_4_ 334.2013; found, 334.2017 (M + H^+^).

#### Synthesis of Products **9a**–**g**

For a reaction on a 2 mmol scale, a solution containing substrate acid **4** (1 M, toluene, 2 mmol, 1.0 equiv) and NEt_3_ (2 mml, 1.0 equiv) was prepared and pumped using a Vaportec E-series flow reactor at a flow rate of 0.25 mL/min. A second solution containing DPPA (0.95 M, toluene, 1.9 mmol, 0.95 equiv) was pumped at the same flow rate and mixed with the substrate solution via a T-piece (1/16 in. id). The combined mixture was then directed into a heated coil reactor (10 mL volume, PFA tubing, 1/16 in. id) held at 100 °C resulting in a residence time of 20 min. Upon exiting this reactor coil, the mixture passed through a BPR (100 psi, Kinesis) before entering an Omnifit glass column containing A-15 and A-21 scavenger resins (mixed bed, ca. 2 equiv each) at an ambient temperature. The crude mixture was collected in a flask and evaporated under reduced pressure. Purification was performed via silica gel chromatography using EtOAc/cyclohexane (10–20% EtOAc) as an eluent system.

A scaled version of this procedure was used starting from acid **4a** (1.55 g, 6.78 mmol) to prepare compound **9a** (1.20 g, 5.97 mmol, 88%).

#### *tert*-Butyl (4-Oxopentyl)carbamate (**9a**).^11^

Yield: 1.20 g (5.97 mmol, 88%). Appearance: colorless oil. ^1^H NMR (400 MHz, CDCl_3_): δ/ppm 4.61 (br s, 1H), 3.08 (q, *J* = 6.6 Hz, 2H), 2.45 (t, *J* = 7.1 Hz, 2H), 2.11 (s, 3H), 1.72 (p, *J* = 7.0 Hz, 2H), 1.40 (s, 9H). ^13^C{^1^H} NMR (100 MHz, CDCl_3_): δ/ppm 208.4 (C), 156.0 (C), 79.1 (C), 40.7 (CH_2_), 39.9 (CH_2_), 29.9 (CH_3_), 28.4 (3CH_3_), 24.2 (CH_2_). IR (neat): ν/cm^–1^ 3365 (br, m), 2976 (m), 2932 (m), 1688 (s), 1517 (m), 1449 (w), 1391 (m), 1365 (s), 1249 (s), 1162 (s). HRMS (TOF-ESI+): calcd for C_10_H_19_NO_3_Na, 224.1257; found, 224.1259 (M + Na^+^).

#### *tert*-Butyl (4-Oxohexyl)carbamate (**9b**)

Yield: 289 mg (1.3 mmol, 84%). Appearance: colorless oil. ^1^H NMR (500 MHz, CDCl_3_): δ/ppm 4.60 (s, 1H), 3.12 (q, *J* = 6.6 Hz, 2H), 2.47–2.41 (m, 4H), 1.77 (p, *J* = 7.0 Hz, 2H), 1.44 (s, 9H), 1.06 (t, *J* = 7.3 Hz, 3H). ^13^C{^1^H} NMR (125 MHz, CDCl_3_): δ/ppm 211.2 (C), 156.0 (C), 79.2 (C), 40.1 (CH_2_), 39.4 (CH_2_), 36.0 (CH_2_), 28.4 (3CH_3_), 24.1 (CH_2_), 7.8 (CH_3_). IR (neat): ν/cm^–1^ 3362 (br), 2976 (m), 2936 (m), 1689 (s), 1517 (m), 1454 (m), 1365 (m), 1248 (s), 1165 (s), 1042 (m), 1023 (m), 980 (m), 875 (m), 780 (m). HRMS (TOF-ESI+): calcd for C_11_H_21_NO_3_Na 238.1414; found, 238.1415 (M + Na^+^).

#### *tert*-Butyl (*E*)-2-Benzylidenepyrrolidine-1-carboxylate (**9c**).^17a^

Yield: 380 mg (1.47 mmol, 88%). Appearance: white solid. Melting range: 78–81 °C. ^1^H NMR (500 MHz, CDCl_3_): δ/ppm 7.32–7.28 (m, 2H), 7.26–7.23 (m, 2H), 7.15–7.11 (m, 2H), 3.66 (t, *J* = 7.0 Hz, 2H), 2.82 (td, *J* = 7.4, 2.0 Hz, 2H), 1.86 (p, *J* = 7.2 Hz, 2H), 1.56 (s, 9H). ^13^C{^1^H} NMR (125 MHz, CDCl_3_): δ/ppm 152.9 (C), 140.9 (C), 139.0 (C), 128.2 (2CH), 128.0 (2CH), 125.0 (CH), 108.3 (CH), 80.6 (C), 48.9 (CH_2_), 30.6 (CH_2_), 28.5 (3CH_3_), 22.1 (CH_2_). IR (neat): ν/cm^–1^ 2975 (w), 2883 (w), 1702 (s), 1639 m), 1383 (s), 1323 (s), 1243 (m), 1159 (s), 1136 (s), 1076 (m), 1003 (m), 856 (m), 748 (m), 694 (s). HRMS (TOF-ESI+): calcd for C_16_H_22_NO_2_, 260.1645; found, 260.1647 (M + H^+^).

#### *tert*-Butyl (*E*)-2-(4-Fluorobenzylidene)pyrrolidine-1-carboxylate (**9d**)

Yield: 223 mg (0.81 mmol, 81%). Appearance: white solid. Melting range: 65–68 °C. ^1^H NMR (400 MHz, CDCl_3_): δ/ppm 7.14 (dd, *J* = 8.7, 5.6 Hz, 2H), 7.06 (br s, 1H), 6.94 (t, *J* = 8.8 Hz, 2H), 3.62 (t, *J* = 7.0 Hz, 2H), 2.72 (td, *J* = 7.4, 2.0 Hz, 2H), 1.82 (p, *J* = 7.2 Hz, 2H), 1.51 (s, 9H). ^13^C{^1^H} NMR (100 MHz, CDCl_3_): δ/ppm 160.6 (CF, d, *J* = 243 Hz), 152.8 (C), 140.7 (C), 134.9 (C, d, *J* = 4 Hz), 129.4 (2CH, d, *J* = 8 Hz), 114.8 (2CH, d, *J* = 21 Hz), 107.2 (CH), 80.6 (C), 48.8 (CH_2_), 30.4 (CH_2_), 28.4 (3CH_3_), 22.0 (CH_2_). ^19^F-NMR (376 MHz, CDCl_3_): δ/ppm δ −118 (s). IR (neat): ν/cm^–1^ 2977 (m), 2934 (w), 1703 (s), 1645 (m), 1507 (s), 1386 (s), 1329 (s), 1228 (m), 1158 (m), 1140 (s), 1002 (m), 860 (m), 768 (m). HRMS (TOF-ESI+): calcd for C_16_H_20_NFO_2_, 278.1551; found, 278.1552 (M + H^+^).

#### *tert*-Butyl (*E*)-2-(4-(Trifluoromethyl)benzylidene)pyrrolidine-1-carboxylate (**9e**)

Yield: 457 mg (1.40 mmol, 85%). Appearance: white solid. Melting range: 86–88 °C. ^1^H NMR (500 MHz, CDCl_3_): δ/ppm δ 7.51 (d, *J* = 8.2 Hz, 2H), 7.30 (d, *J* = 8.2 Hz, 2H), 7.16 (br s, 1H), 3.66 (t, *J* = 6.9 Hz, 2H), 2.81 (td, *J* = 7.3, 2.0 Hz, 2H), 1.87 (p, *J* = 7.2 Hz, 2H), 1.54 (s, 9H). ^13^C{^1^H} NMR (125 MHz, CDCl_3_): δ/ppm 152.7 (C), 143.0 (C), 142.8 (C), 128.0 (2CH), 126.6 (C, q, *J* = 32.3 Hz), 124.9 (2CH, q, *J* = 3.9 Hz), 124.5 (CF_3_, q, *J* = 270 Hz), 107.0 (CH), 81.0 (C), 49.0 (CH_2_), 30.7 (CH_2_), 28.3 (3CH_3_), 22.0 (CH_2_). ^19^F-NMR (282 MHz, CDCl_3_): δ/ppm δ −62.2 (s). IR (neat): ν/cm^–1^ 2978 (w), 2889 (w), 1706 (m), 1637 (m), 1610 (m), 1386 (m), 1314 (s), 1244 (m), 1159 (s), 1140 (s), 1107 (s), 1066 (s), 1002 (m), 859 (s). HRMS (TOF-ESI+): calcd for C_17_H_20_F_3_NO_2_Na, 350.1338; found, 350.1338 (M + Na^+^). Crystal data: CCDC 2083011: C_17_H_20_F_3_NO_2_; monoclinic, *a* = 15.3119(2) Å, *b* = 10.2080(2) Å, *c* = 21.3592(3) Å, α = 90°, β = 92.8390(10)°, γ = 90°; *Z* = 8; space group *P*2_1_/*c*; *T* = 100 K; *R*_1_ = 0.0872.

#### *tert*-Butyl (4-Oxo-7-phenylheptyl)carbamate (**9g**)

Yield: 238 mg (0.79 mmol, 79%). Appearance: clear oil. ^1^H NMR (500 MHz, CDCl_3_): δ/ppm 7.28 (t, *J* = 7.7 Hz, 2H), 7.22–7.14 (m, 3H), 4.58 (s, 1H), 3.10 (q, *J* = 6.7 Hz, 2H), 2.61 (t, *J* = 7.6 Hz, 2H), 2.41 (m, 4H), 1.90 (p, *J* = 7.4 Hz, 2H), 1.73 (p, *J* = 7.0 Hz, 2H), 1.43 (s, 9H). ^13^C{^1^H} NMR (125 MHz, CDCl_3_): δ/ppm 210.3 (C), 156.0 (C), 141.5 (C), 128.5 (2CH), 128.4 (2CH), 125.9 (CH), 79.2 (C), 42.0 (CH_2_), 40.0 (CH_2_), 39.9 (CH_2_), 35.1 (CH_2_), 28.4 (3CH_3_), 25.2 (CH_2_), 24.0 (CH_2_). IR (neat): ν/cm^–1^ 3368 (br, m), 2975 (m), 2931 (m), 1701 (s), 1513 (m), 1453 (m), 1391 (m), 1365 (m), 1248 (m), 1165 (s), 748 (m), 700 (s). HRMS (TOF-ESI+): calcd for C_18_H_27_NO_3_Na, 328.1883; found, 328.1886 (M + Na^+^).

## Supporting Information Available

Copies of ^1^H and ^13^C NMR spectra (PDF)

## Accession Codes

CCDC 2083011 contains the supplementary crystallographic data for this paper. These data can be obtained free of charge via www.ccdc.cam.ac.uk/data_request/cif, or by emailing data_request@ccdc.cam.ac.uk, or by contacting The Cambridge Crystallographic Data Centre, 12 Union Road, Cambridge CB2 1EZ, UK; fax: +44 1223 336033.

## References

[ref1] aCurtius T. Ueber Stickstoffwasserstoffsäure (Azoimid) N_3_H. Ber. Dtsch. Chem. Ges. 1890, 23, 3023–3033. 10.1002/cber.189002302232.

[ref2] aThomas M.; Alsarraf J.; Araji N.; Tranoy-Opalinski I.; Renoux B.; Papot S. The Lossen rearrangement from free hydroxamic acids. Org. Biomol. Chem. 2019, 17, 5420–5427. 10.1039/C9OB00789J. 31090777

[ref3] aWentrup C.; Bornemann H. The Curtius Rearrangement of Acyl Azides Revisited - Formation of Cyanate (R-O-CN). Eur. J. Org. Chem. 2005, 2005, 4521–4524. 10.1002/ejoc.200500545.

[ref4] aGhosh A. K.; Brindisi M.; Sarkar A. The Curtius Rearrangement: Applications in Modern Drug Discovery and Medicinal Chemistry. ChemMedChem 2018, 13, 2351–2373. 10.1002/cmdc.201800518. 30187672 PMC6604631

[ref5] aGuidi M.; Seeberger P. H.; Gilmore K. How to approach flow chemistry. Chem. Soc. Rev. 2020, 49, 8910–8932. 10.1039/C9CS00832B. 33140749

[ref6] aSahoo H. R.; Kralj J. G.; Jensen K. F. Multistep Continuous-Flow Microchemical Synthesis Involving Multiple Reactions and Separations. Angew. Chem., Int. Ed. 2007, 46, 5704–5708. 10.1002/anie.200701434. 17579912

[ref7] aHuard K.; Bagley S. W.; Menhaji-Klotz E.; Preville C.; Southers J. A. Jr; Smith A. C.; Edmonds D. J.; Lucas J. C.; Dunn M. F.; Allanson N. M.; Blaney E. L.; Garcia-Irizarry C. N.; Kohrt J. T.; Griffith D. A.; Dow R. L. Synthesis of spiropiperidine lactam acetyl-CoA carboxylase inhibitors. J. Org. Chem. 2012, 77, 10050–10057. 10.1021/jo3014808. 23127254

[ref8] Marsini M. A.; Buono F. G.; Lorenz J. C.; Yang B.-S.; Reeves J. T.; Sidhu K.; Sarvestani M.; Tan Z.; Zhang Y.; Li N.; Lee H.; Brazzillo J.; Nummy L. J.; Chung J. C.; Luvaga I. K.; Narayanan B. A.; Wei X.; Song J. J.; Roschangar F.; Yee N. K.; Senanayake C. H. Development of a concise, scalable synthesis of a CCR1 antagonist utilizing a continuous flow Curtius rearrangement. Green Chem. 2017, 19, 1454–1461. 10.1039/C6GC03123D.

[ref9] aLeslie A.; Moody T. S.; Smyth M.; Wharry S.; Baumann M. Coupling biocatalysis with high-energy flow reactions for the synthesis of carbamates and β-amino acid derivatives. Beilstein J. Org. Chem. 2021, 17, 379–384. 10.3762/bjoc.17.33. 33828617 PMC7871027

[ref10] Substrate **4a** was obtained from alkaline hydrolysis of methyl ester **11a**, which was kindly provided by Almac Sciences.

[ref11] Lima F.; Sharma U. K.; Grunenberg L.; Saha D.; Johannsen S.; Sedelmeier J.; Van der Eycken E. V.; Ley S. V. A Lewis Base Catalysis Approach for the Photoredox Activation of Boronic Acids and Esters. Angew. Chem., Int. Ed. 2017, 56, 15136–15140. 10.1002/anie.201709690. PMC5708277 29024307

[ref12] aWegner J.; Ceylan S.; Kirschning A. Ten key issues in modern flow chemistry. Chem. Commun. 2011, 47, 4583–4592. 10.1039/c0cc05060a. 21409184

[ref13] Backpressure regulators (100 psi) were purchased from Kinesis (https://kinesis.co.uk/).

[ref14] Amberlyst A-15 and Amberlyst A-21 resins were purchased from Sigma-Aldrich and used after washing with water and methanol.

[ref15] Paryzek Z.; Koenig H.; Tabaczka B. Ammonium Formate/Palladium on Carbon: A Versatile System for Catalytic Hydrogen Transfer Reductions of Carbon-Carbon Double Bonds. Synthesis 2003, 2023–2026. 10.1055/s-2003-41024.

[ref16] This X-ray structure has been deposited as CCDC 2083011 with the Cambridge Crystallographic Data Centre and is freely available from https://www.ccdc.cam.ac.uk/.

[ref17] aCostello J. P.; Ferreira E. M. Regioselectivity Influences in Platinum-Catalyzed Intramolecular Alkyne O-H and N-H Additions. Org. Lett. 2019, 21, 9934–9939. 10.1021/acs.orglett.9b03557. 31815495 PMC9305993

[ref18] aMaryanoff B. E.; Zhang H.-C.; Cohen J. H.; Turchi I. J.; Maryanoff C. A. Cyclizations of N-Acyliminium Ions. Chem. Rev. 2004, 104, 1431–1628. 10.1021/cr0306182. 15008627

[ref19] Subsequent experiments showed that hydrolysis of products **9c**–**e** proceeds slowly in refluxing in MeOH (containing small amounts of HOAc) with ca. 20% conversion over 3 h; longer reaction times led to partial decomposition.

[ref20] Omelian T. V.; Dobrydnev A. V.; Ostapchuk E. N.; Volovenko Y. M. Synthesis of Novel 3a-Substituted Tetrahydro-1H-1λ^6^-pyrrolo[1,2-b]isothiazole-1,1,3(2H)-triones through the CSIC Reaction. ChemistrySelect 2019, 4, 4933–4937. 10.1002/slct.201900650.

[ref21] Kelleher F.; Kelly S.; Watts J.; McKee V. Structure-reactivity relationships of l-proline derived spirolactams and α-methyl prolinamide organocatalysts in the asymmetric Michael addition reaction of aldehydes to nitroolefins. Tetrahedron 2010, 66, 3525–3536. 10.1016/j.tet.2010.03.002.

