# Supplementary material for: Interrupted Curtius Rearrangements of Quaternary Proline Derivatives: A Flow Route to Acyclic Ketones and Unsaturated Pyrrolidines
Source: J Org Chem. 2021 Jun 25;86(20):14199–206. doi: 10.1021/acs.joc.1c01133 (PMC8524412; doi:10.1021/acs.joc.1c01133)

Supporting Information

Interrupted Curtius Rearrangements of Quaternary Proline Derivatives: A Flow Route to Acyclic Ketones and Unsaturated Pyrrolidines

Marcus Baumann,[a,*] Thomas S. Moody,[b,c] Megan Smyth,[b] and Scott Wharry[b]

[a] School of Chemistry, University College Dublin, Science Centre South, Belfield, D04 N2E2, Ireland.
[b] Almac Group Ltd., Craigavon BT63 5QD, United Kingdom.
[c] Arran Chemical Company, Roscommon N37 DN24, Ireland.

Table of Contents:

1. Copies of NMR Spectra SI-2

   a. Methyl esters **11a-11g** SI-2
   b. Acids **4a-g** SI-10
   c. Interrupted Curtius products **9a-9g** SI-17

1. Copies of NMR Spectra

^{1}H and ^{13}C{^{1}H} Spectra of Compound **11b**:

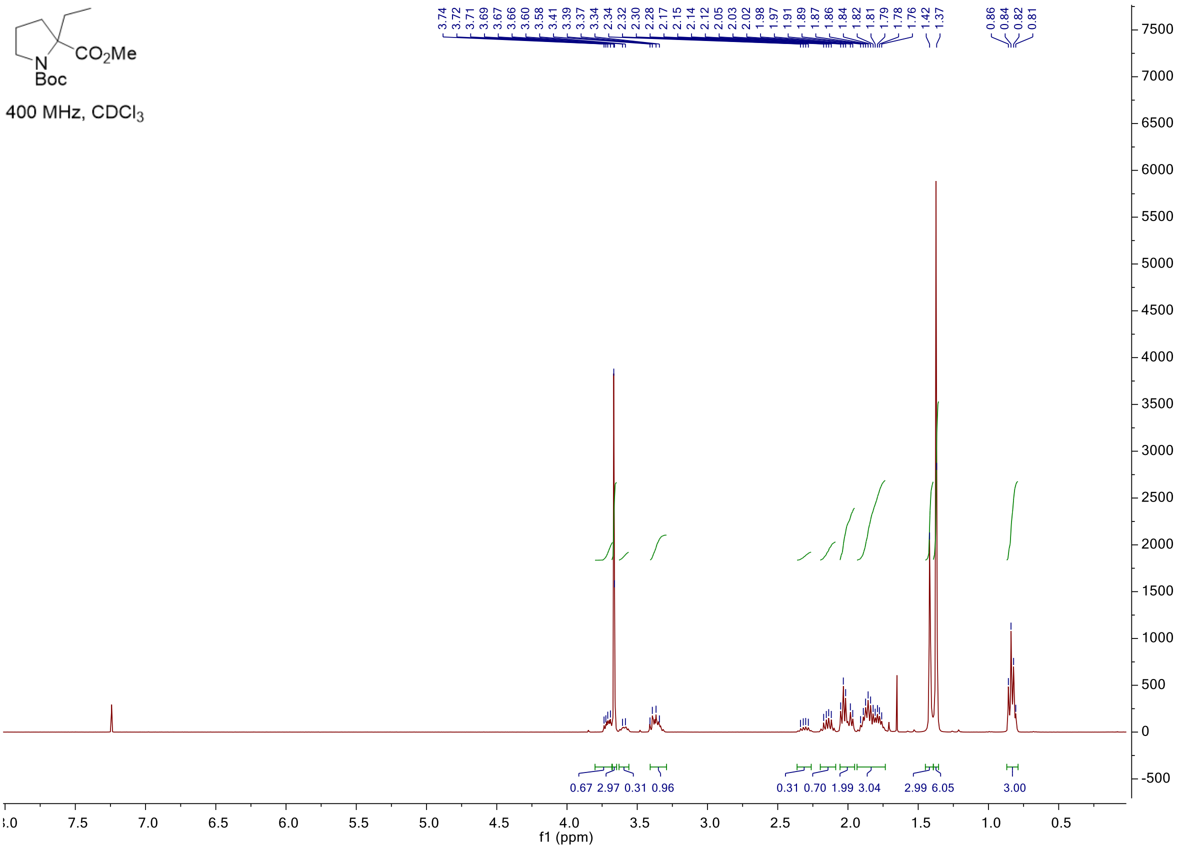

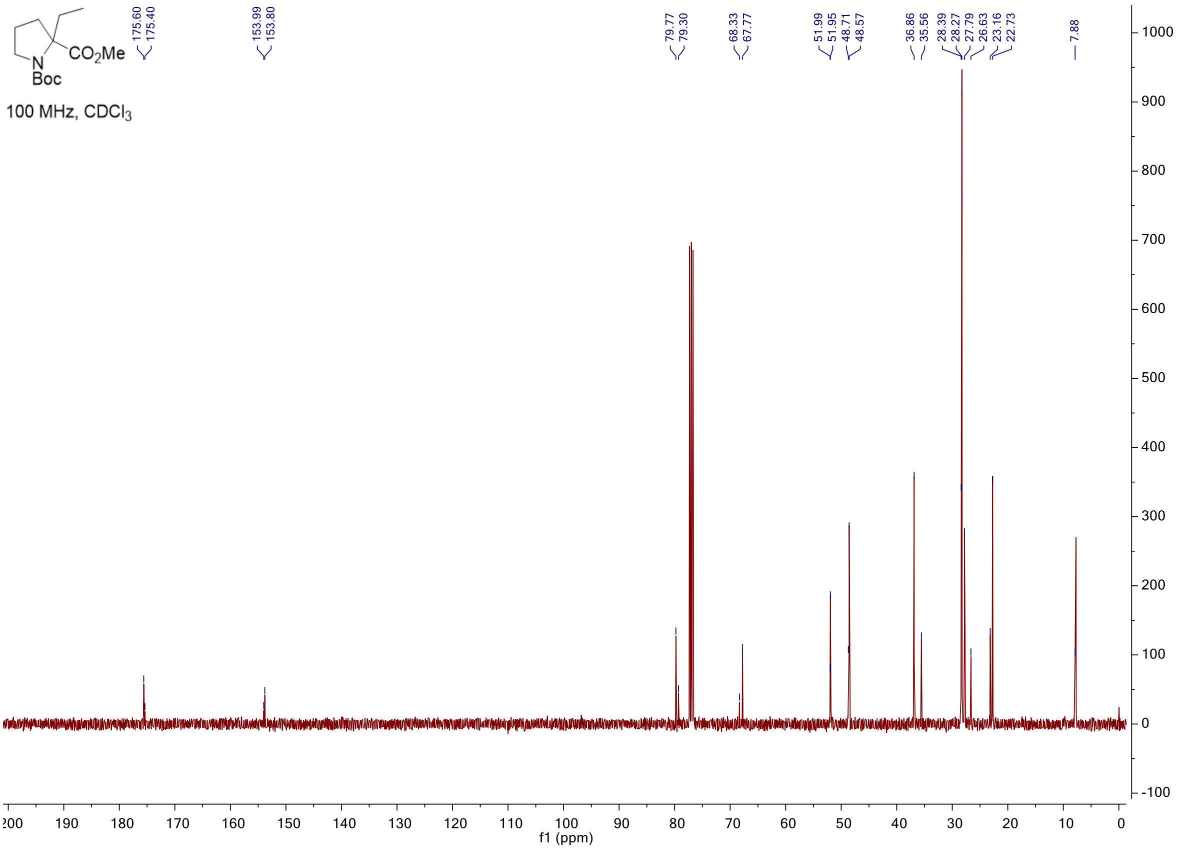

^{1}H and ^{13}C{^{1}H} Spectra of Compound **11c**:

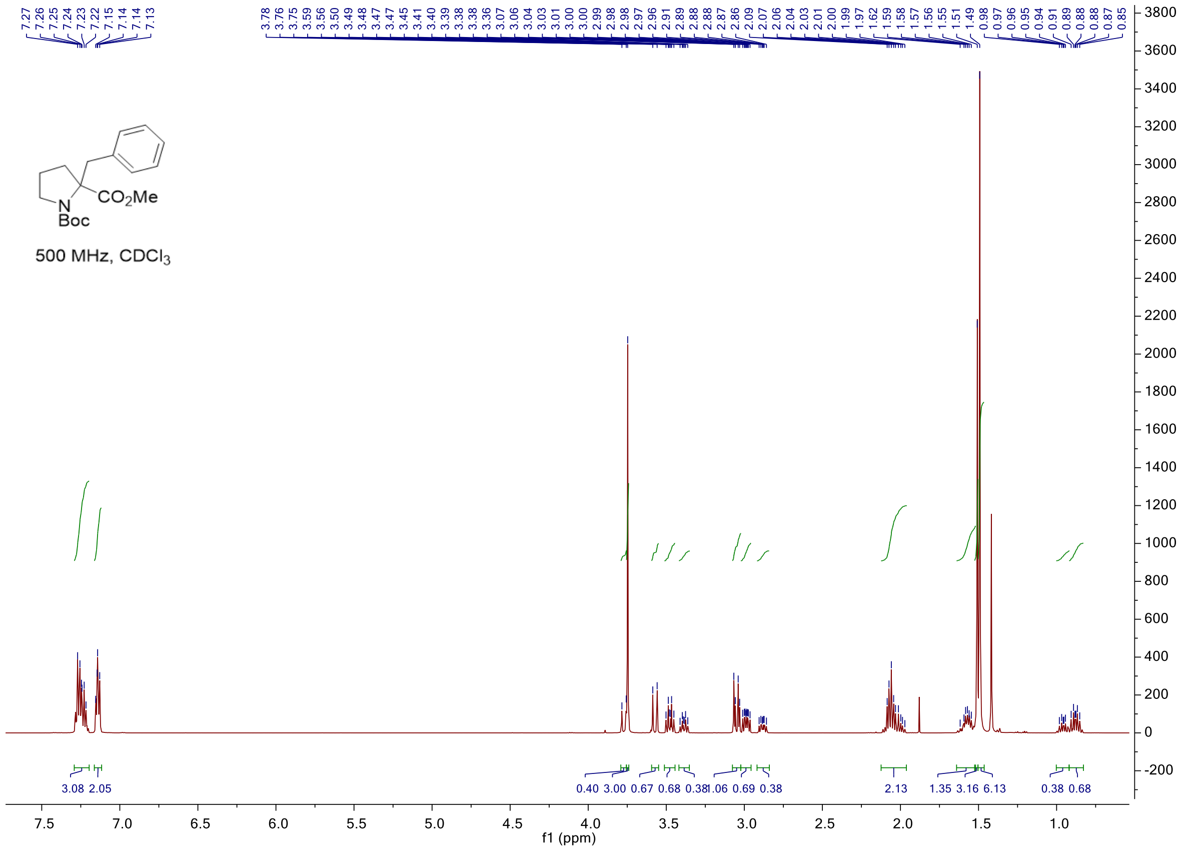

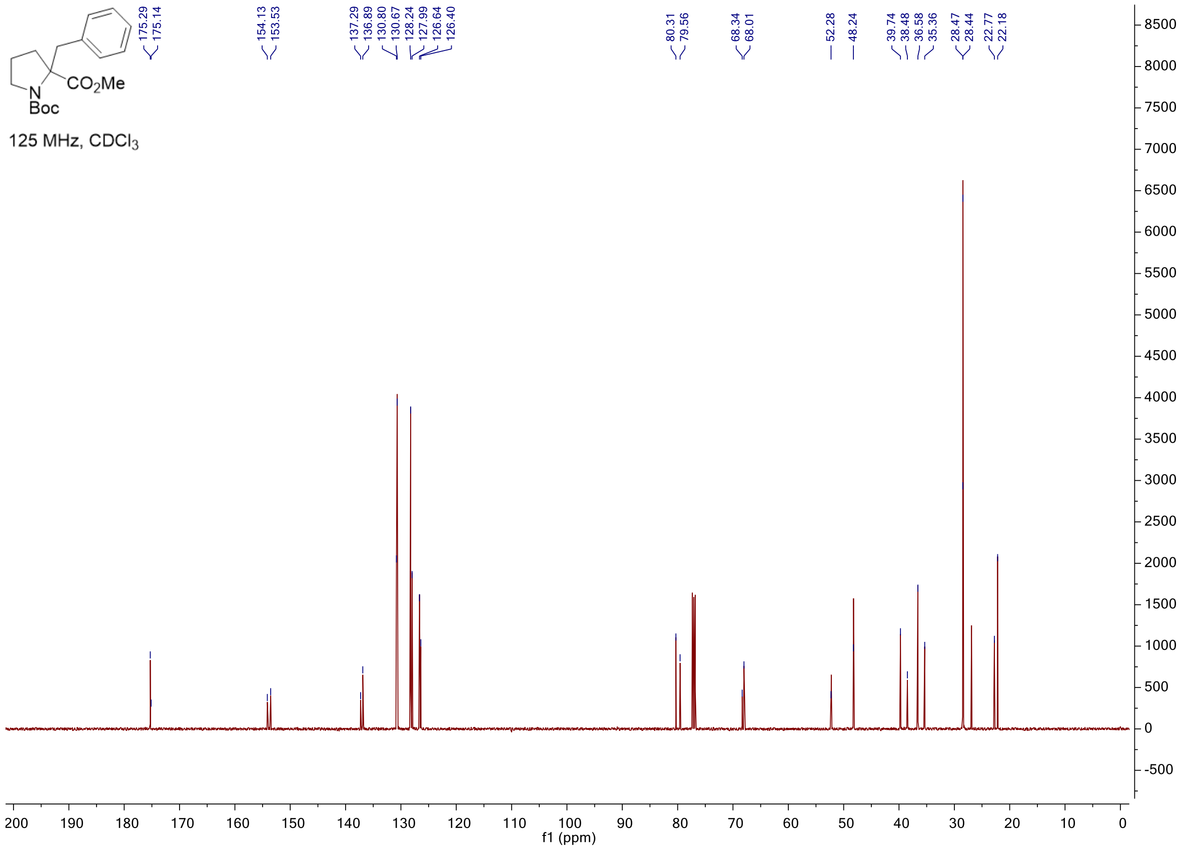

^{1}H, ^{19}F and ^{13}C{^{1}H} Spectra of Compound **11d**:

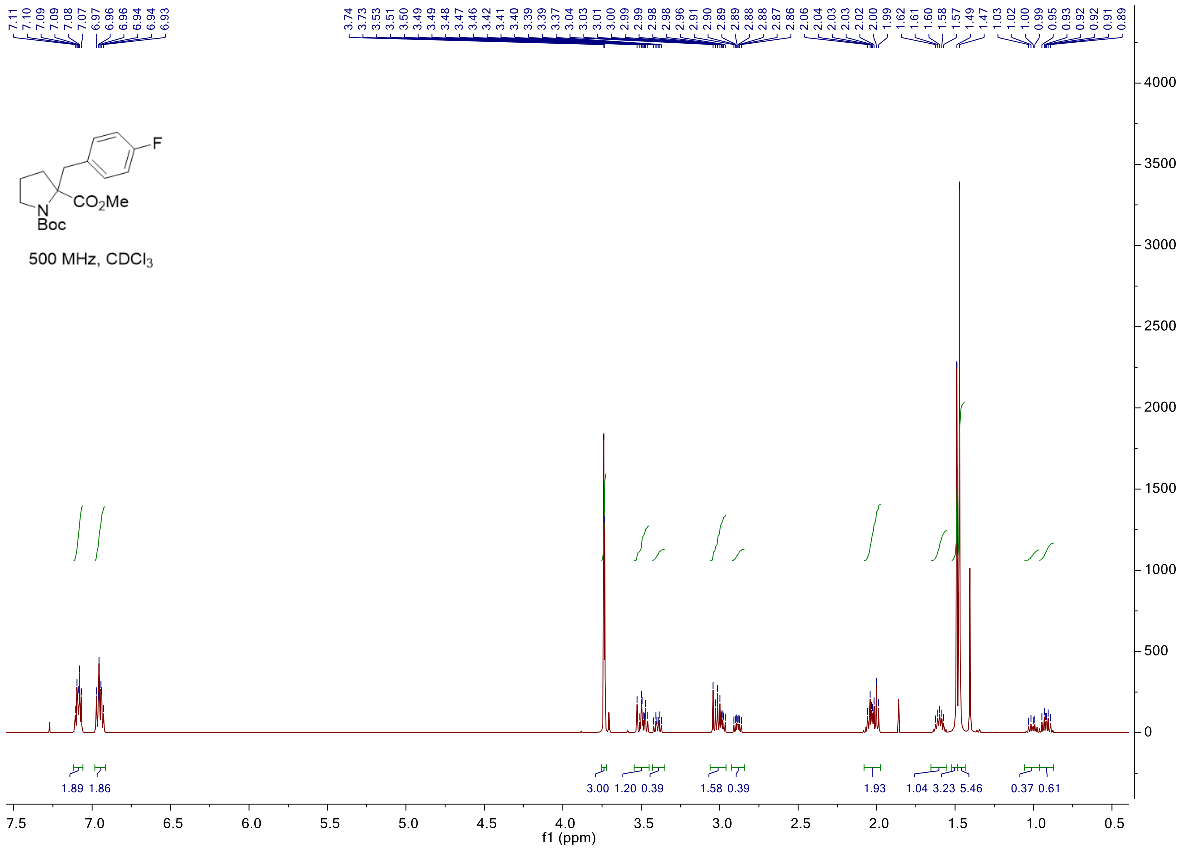

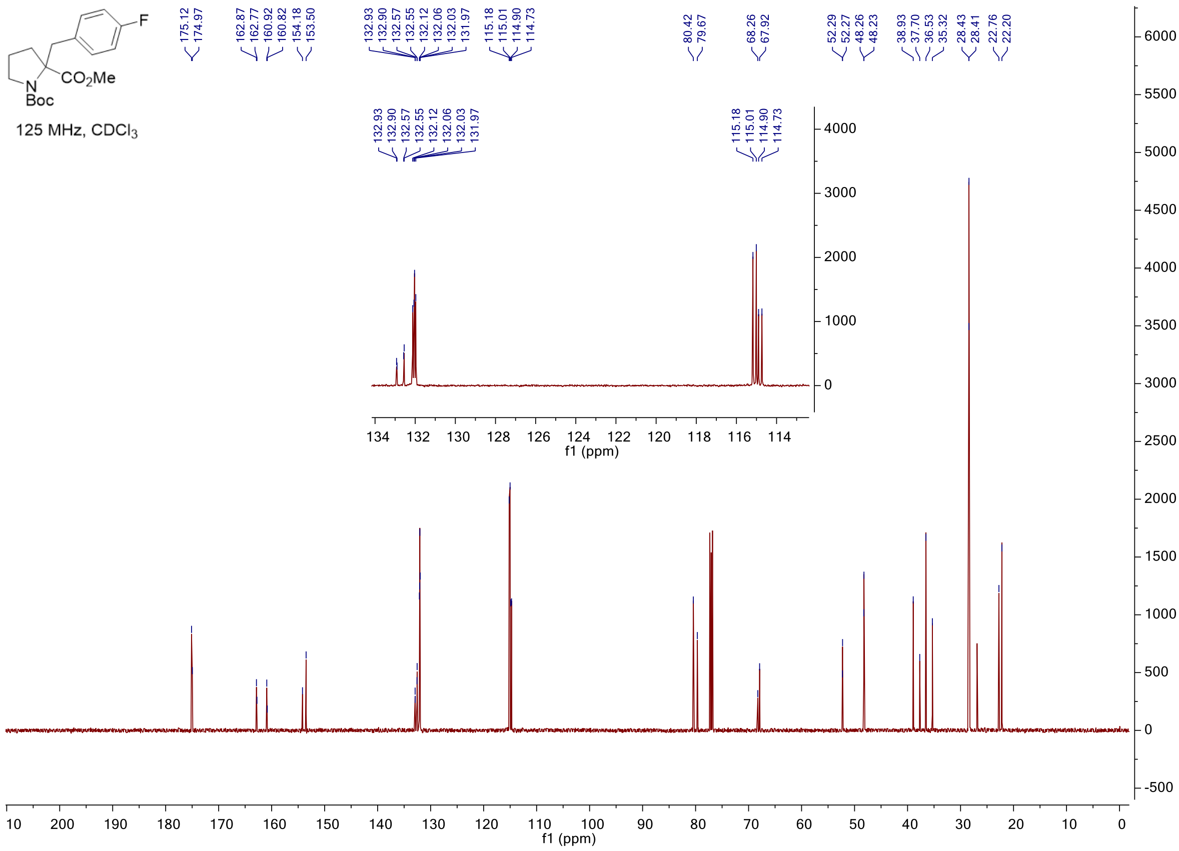

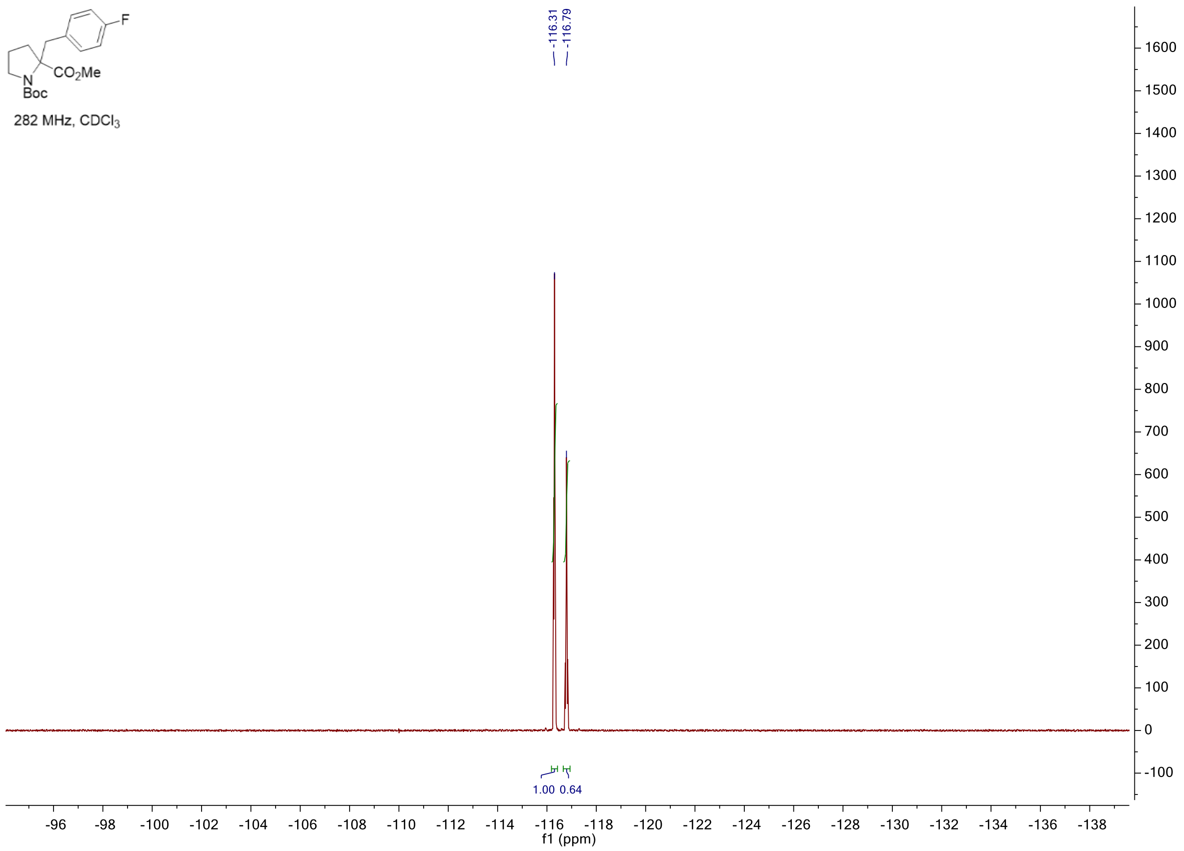
F
CO2Me
N
Boc
282 MHz, CDCl3
-116.31
-116.79
1.00
0.64
f1 (ppm)

^{1}H, ^{19}F and ^{13}C{^{1}H} Spectra of Compound **11e**:

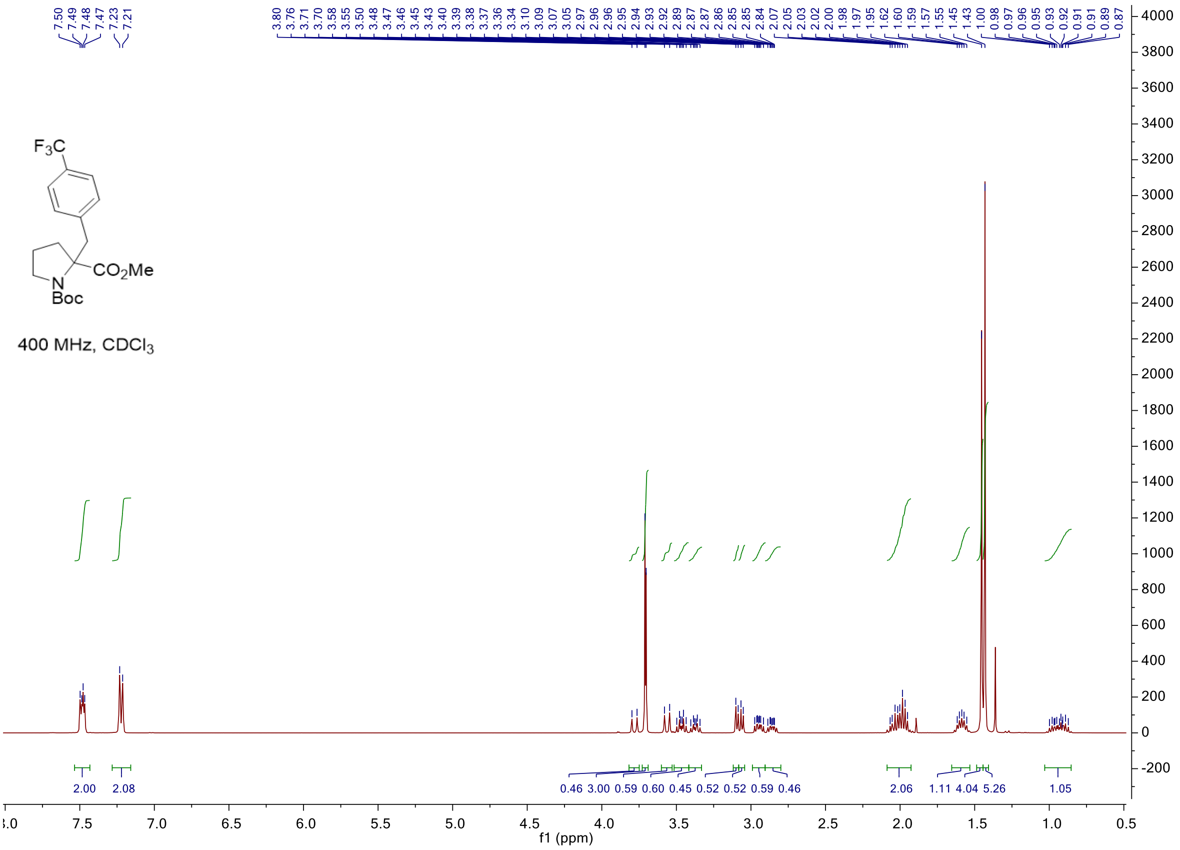

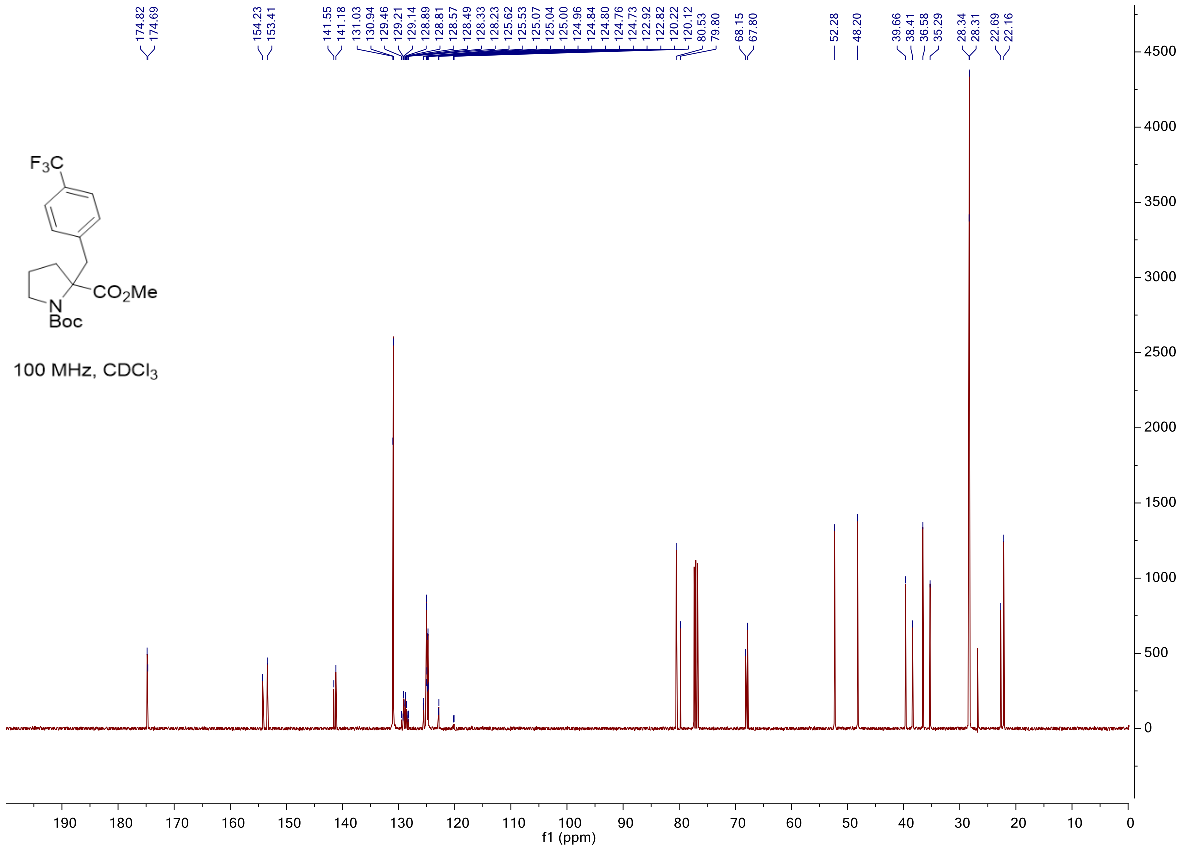

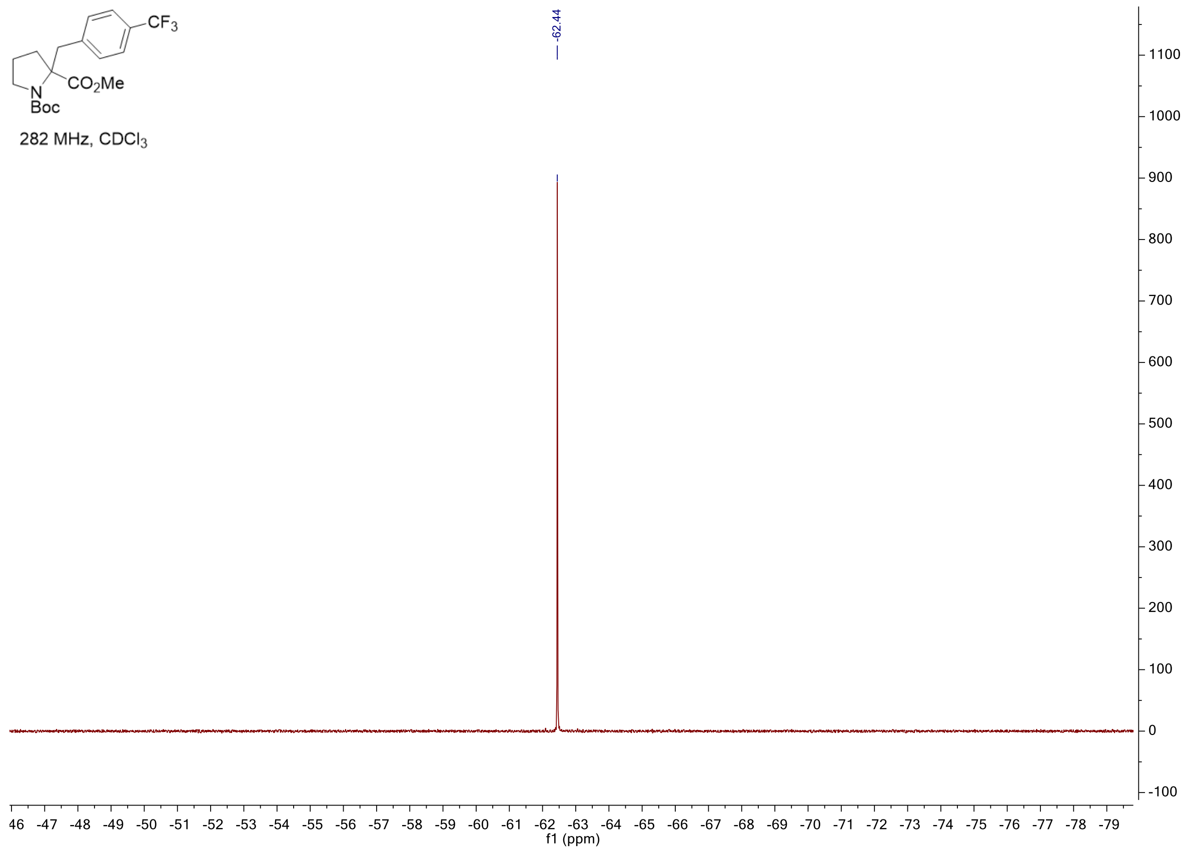
CF3
CO2Me
N
Boc
282 MHz, CDCl3
-62.44
f1 (ppm)

^{1}H and ^{13}C{^{1}H} Spectra of Compound **11f**:

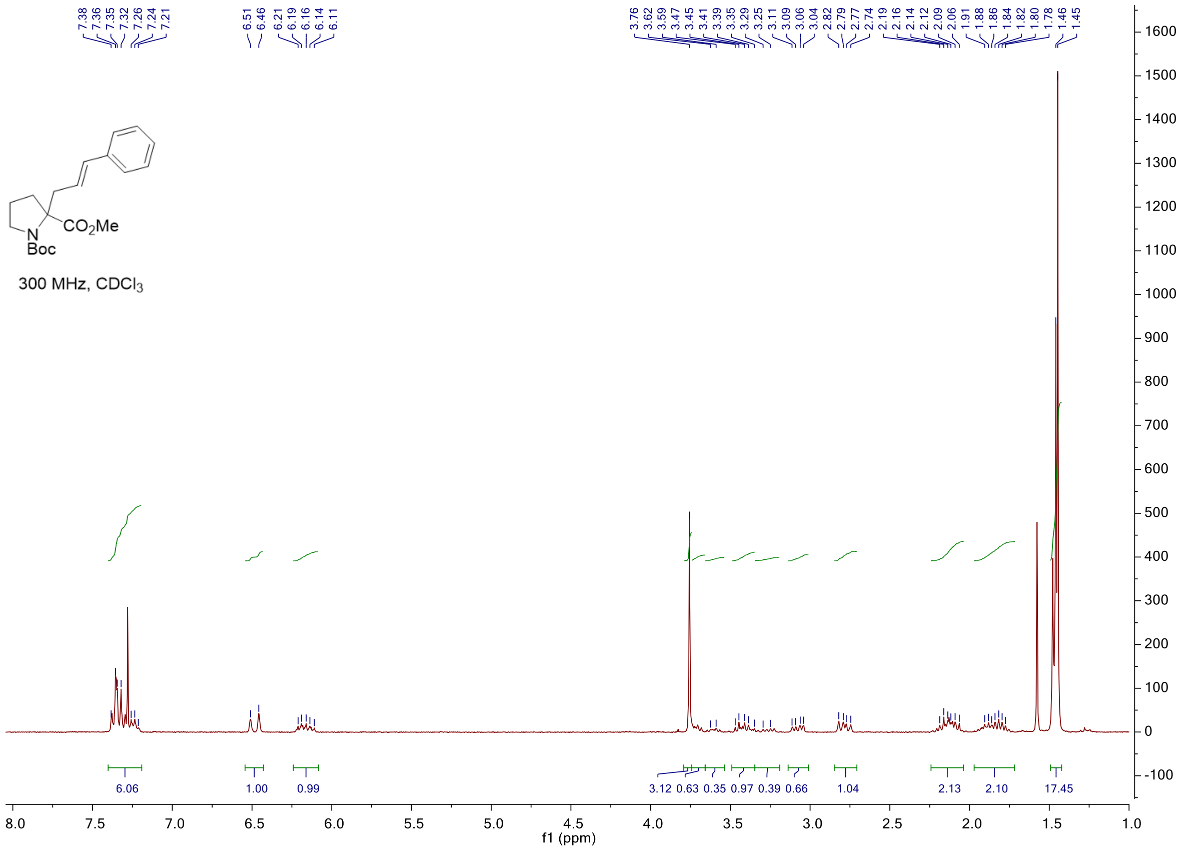

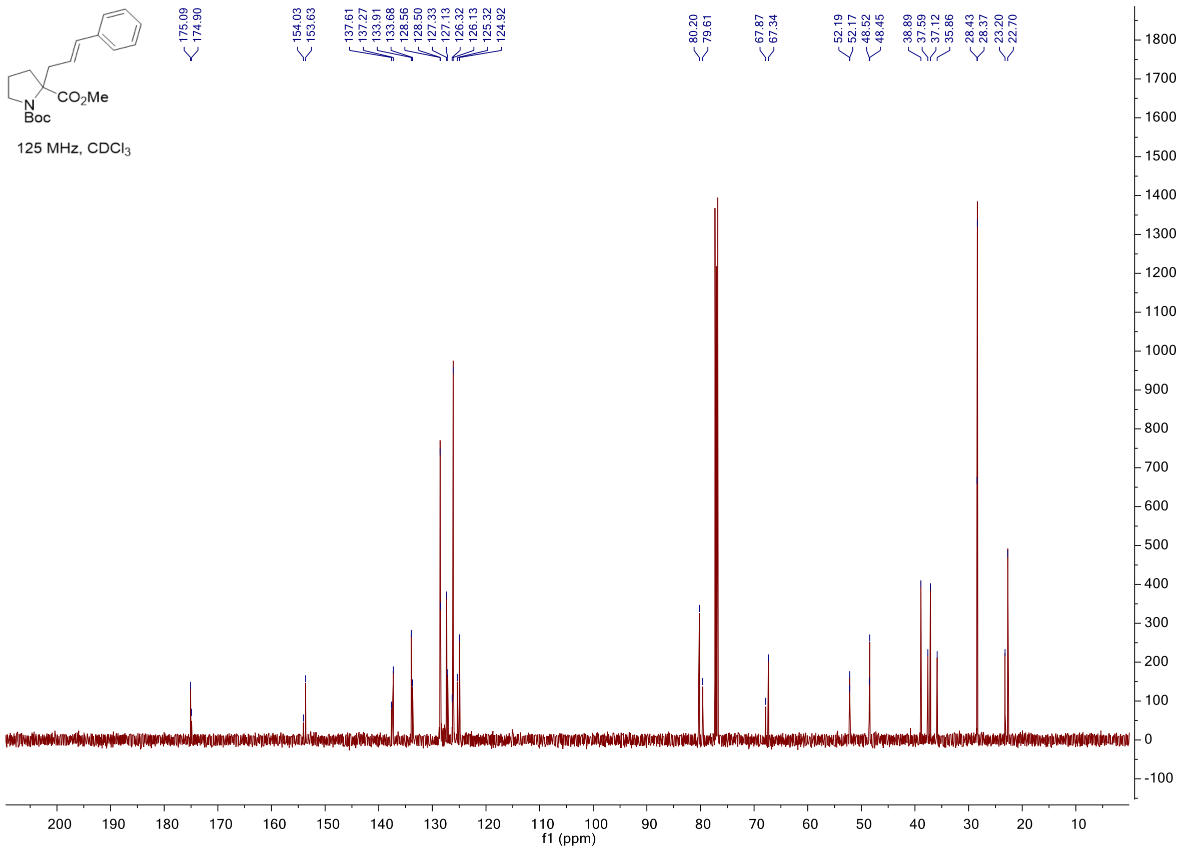

^{1}H and ^{13}C{^{1}H} Spectra of Compound **11g**:

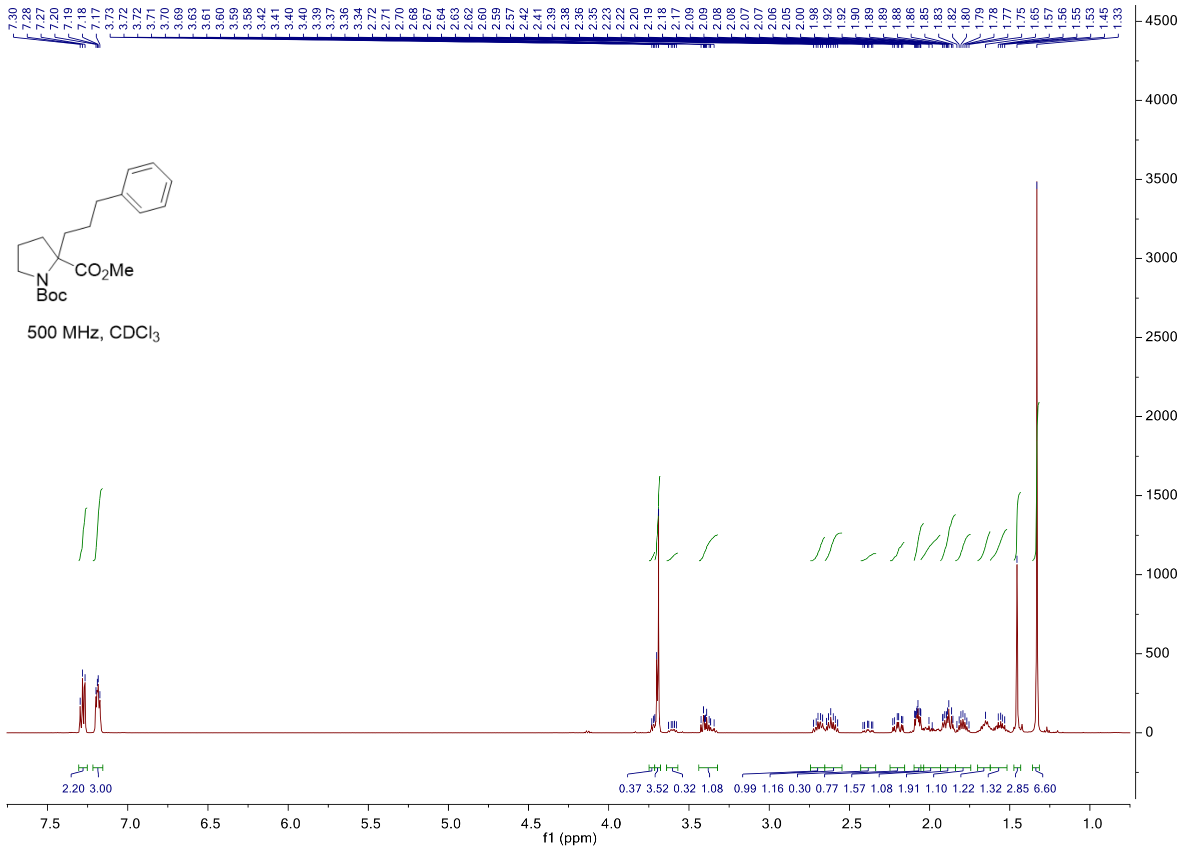

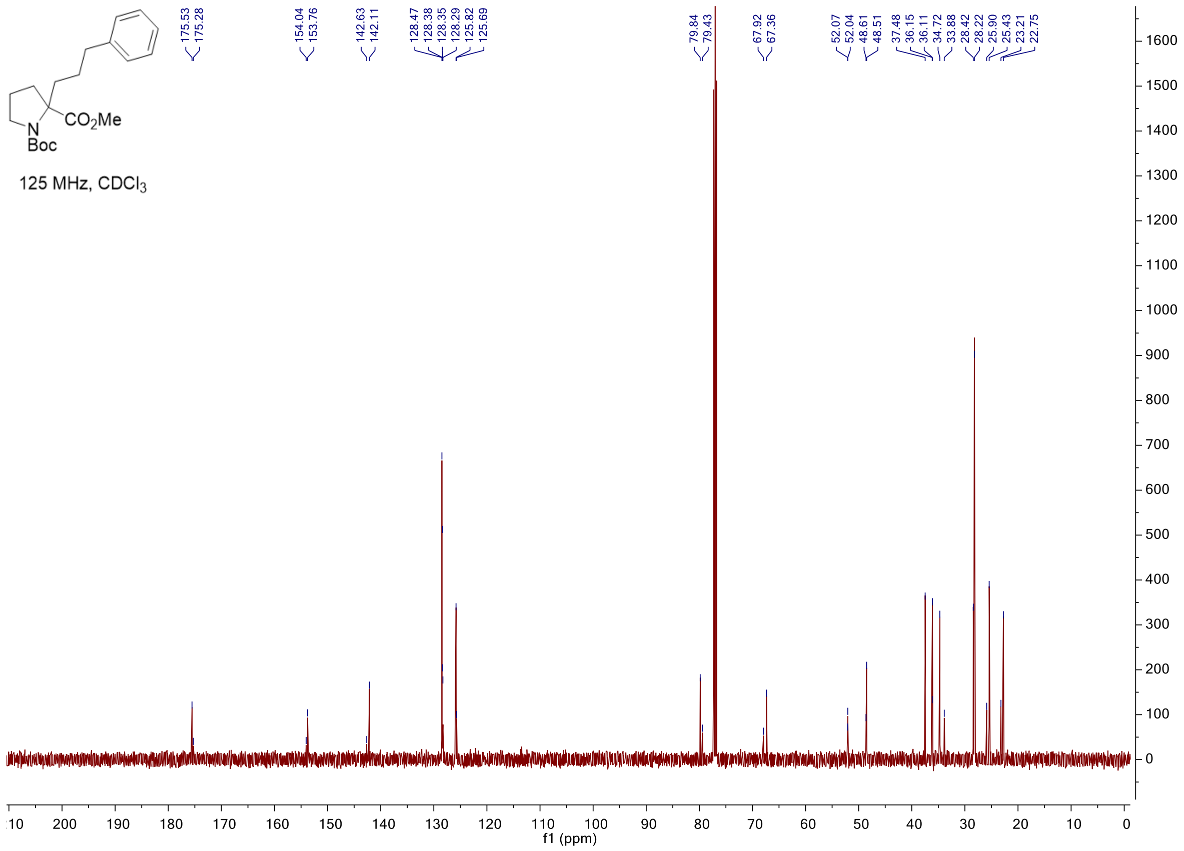

^{1}H and ^{13}C{^{1}H} Spectra of Compound **4a**:

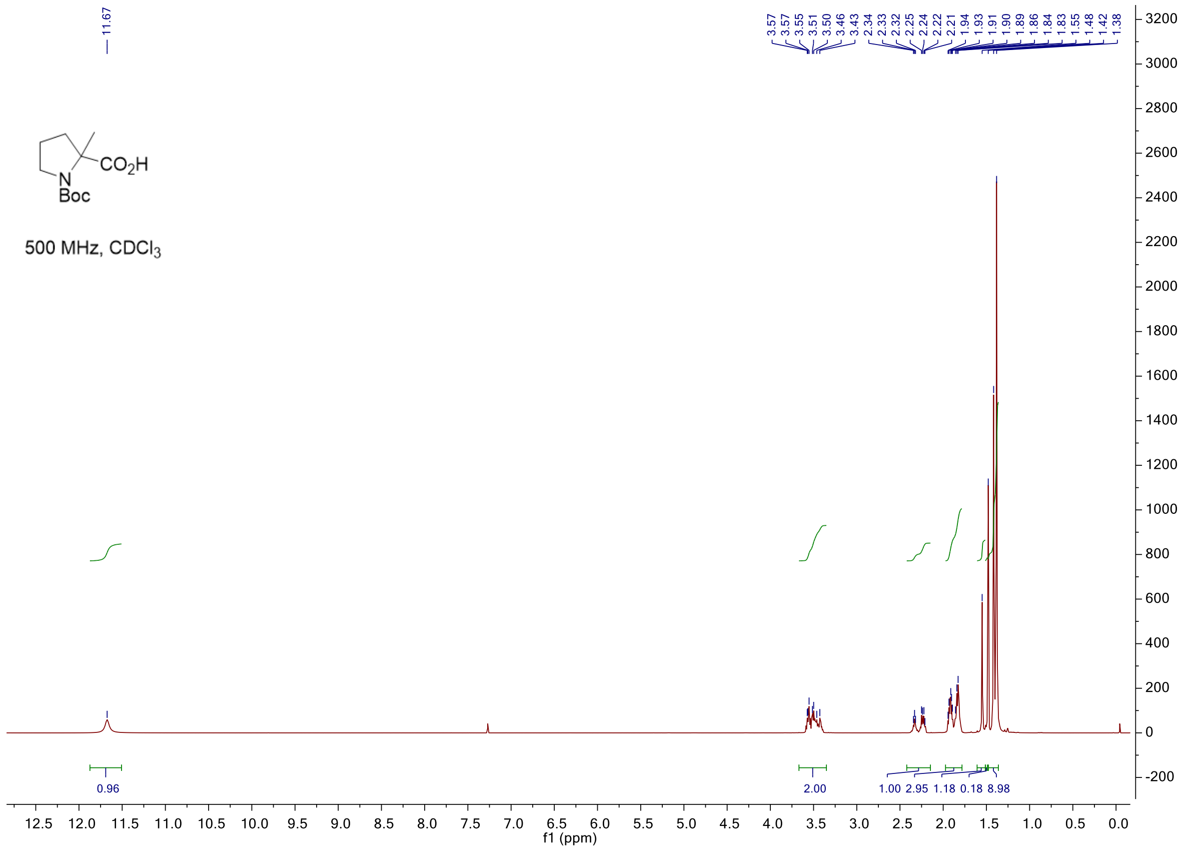

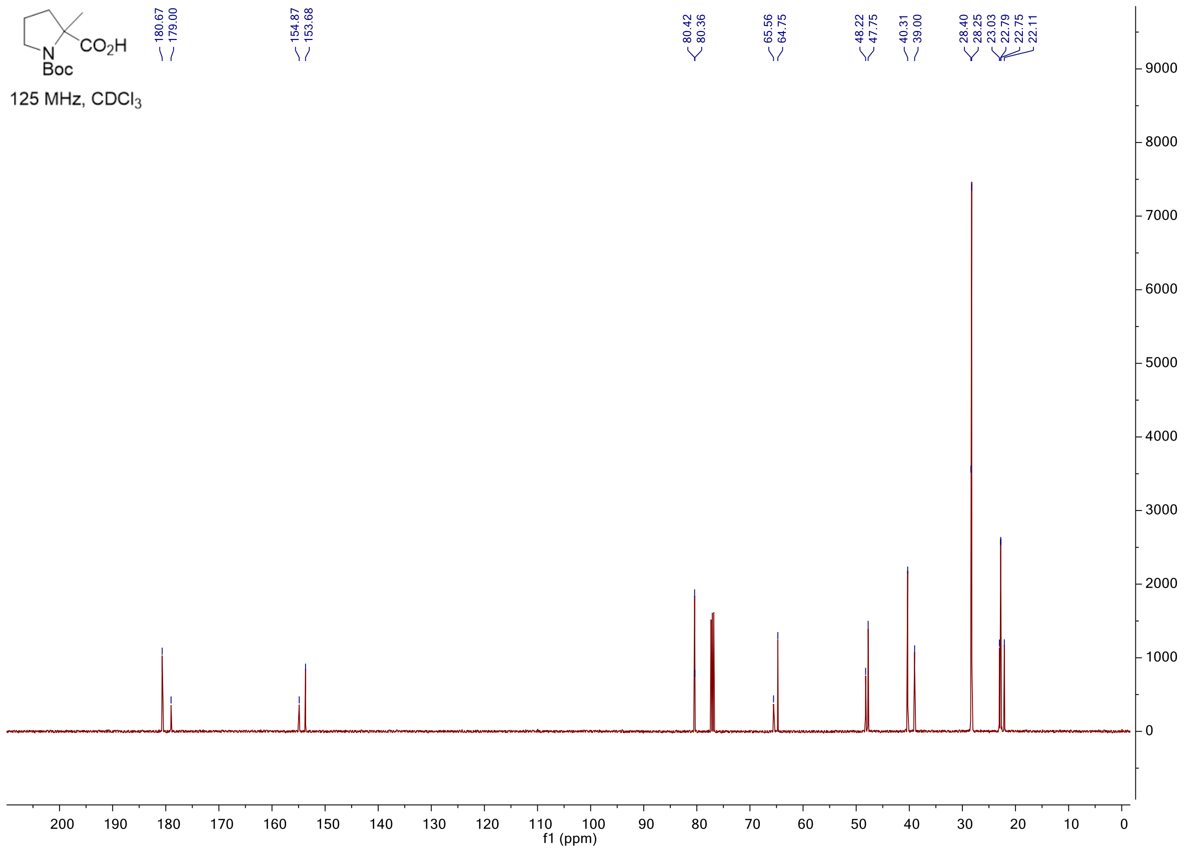

^{1}H and ^{13}C{^{1}H} Spectra of Compound **4b**:

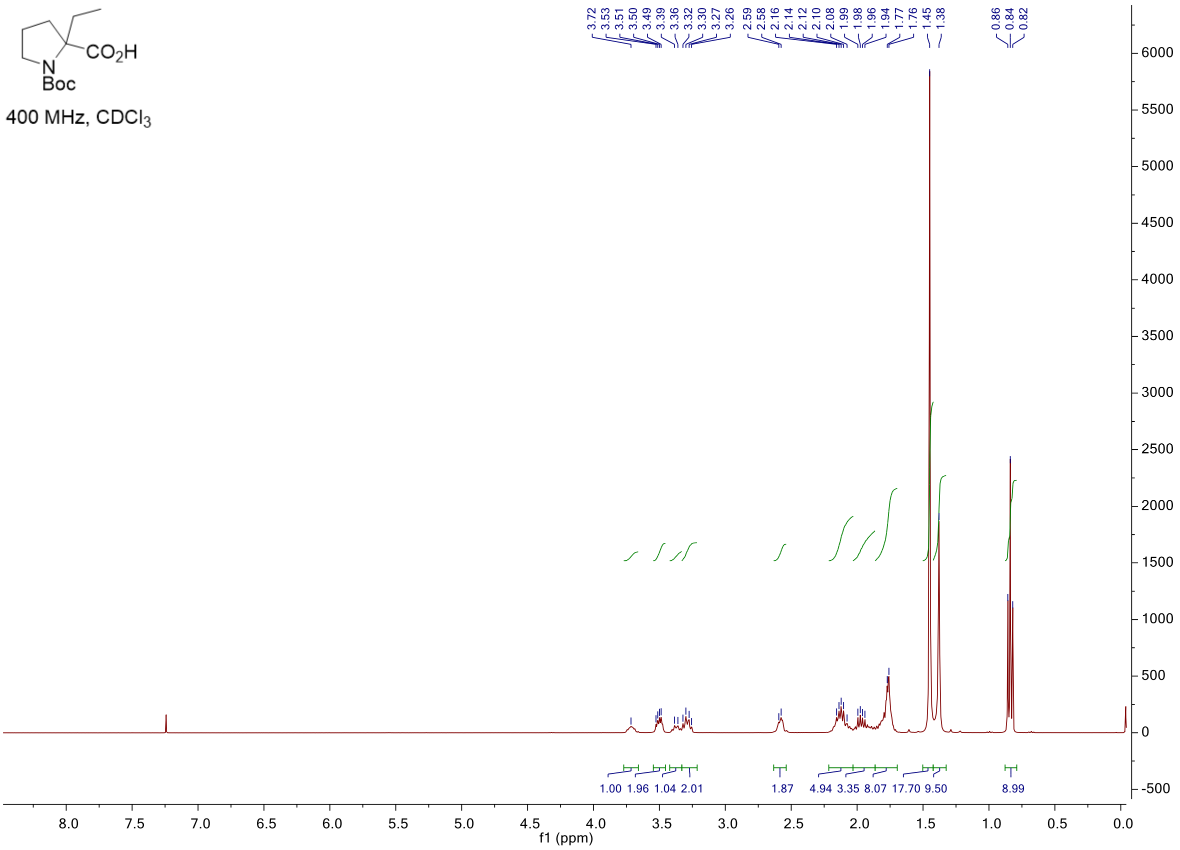

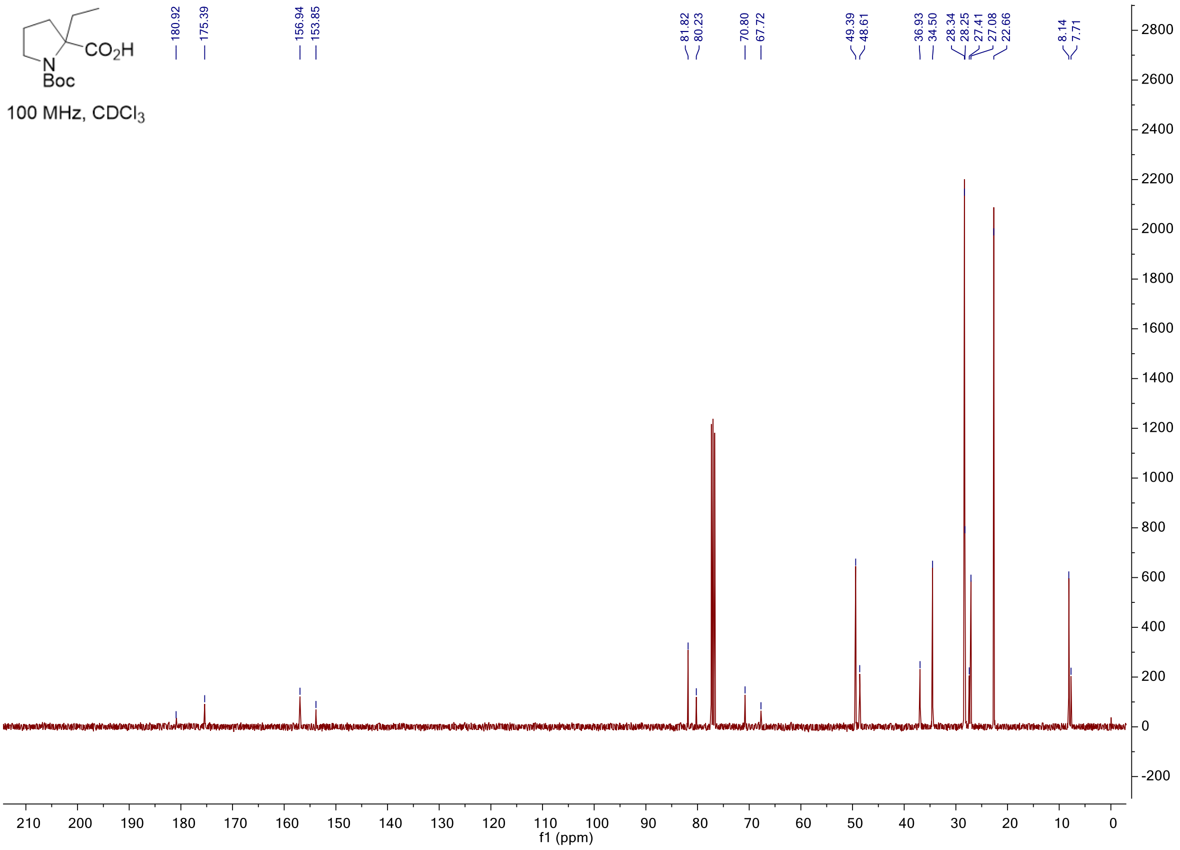

^{1}H and $^{13}C\{^{1}H\}$ Spectra of Compound **4c**:

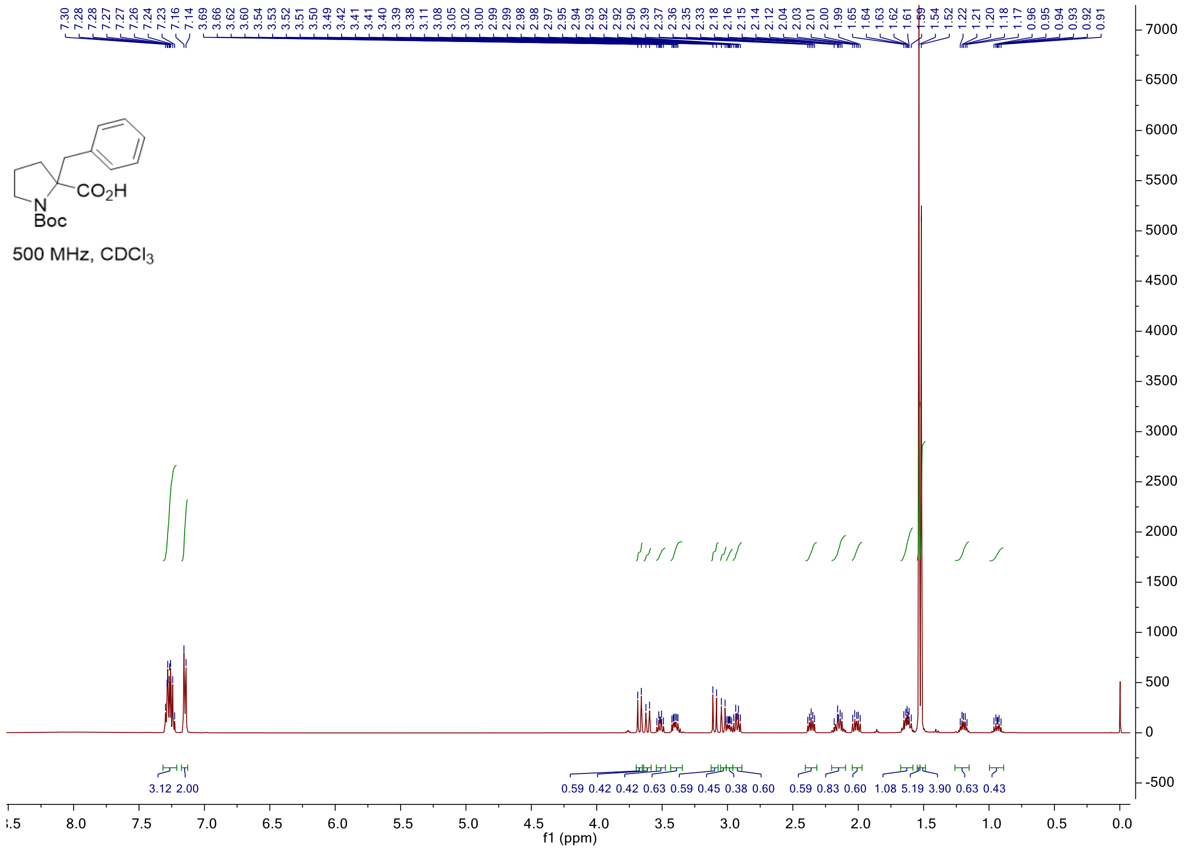

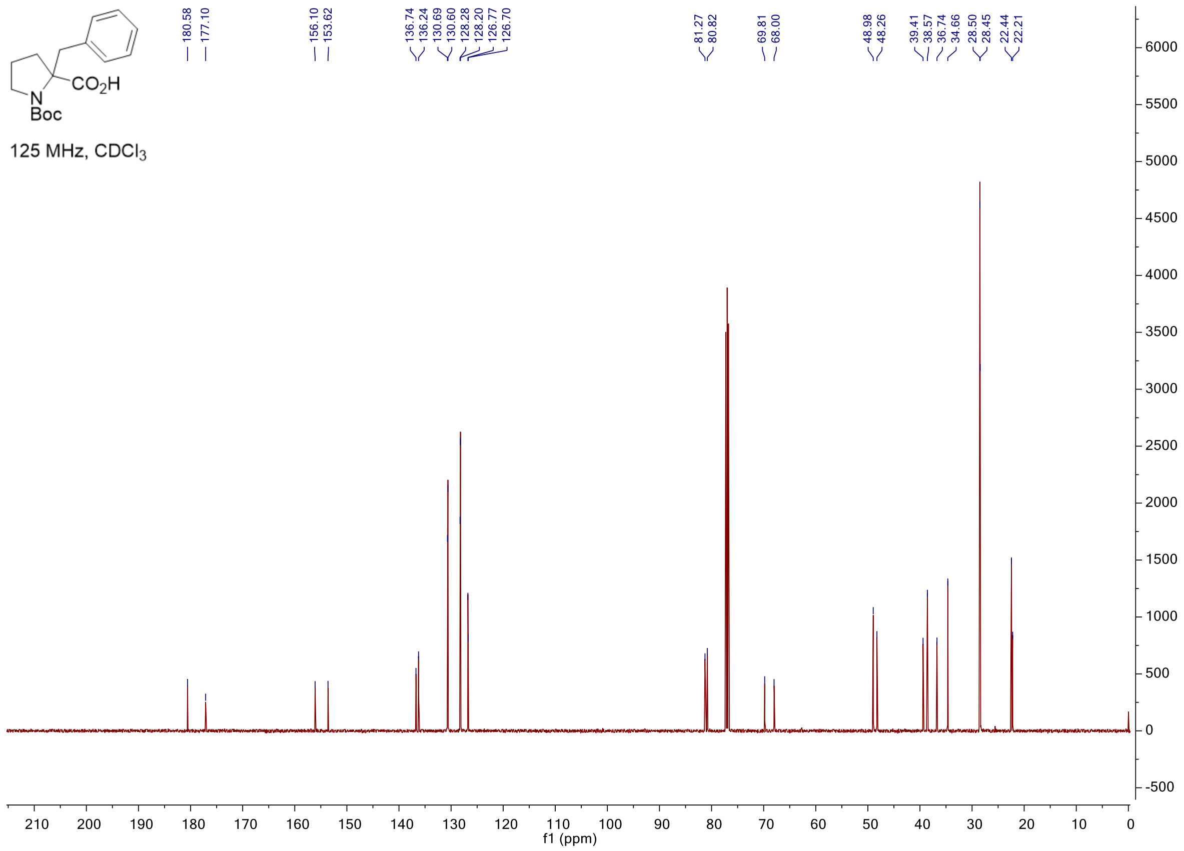

^{1}H and ^{13}C{^{1}H} Spectra of Compound **4d**:

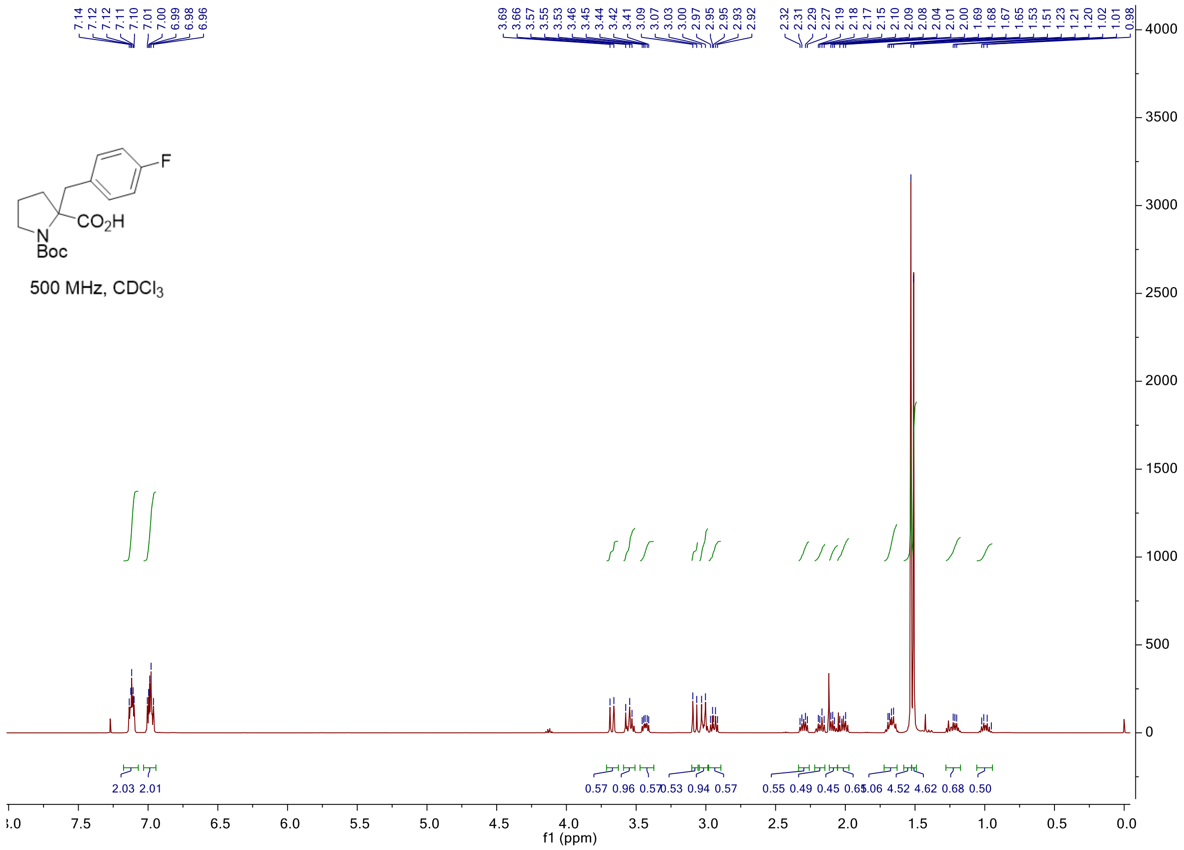

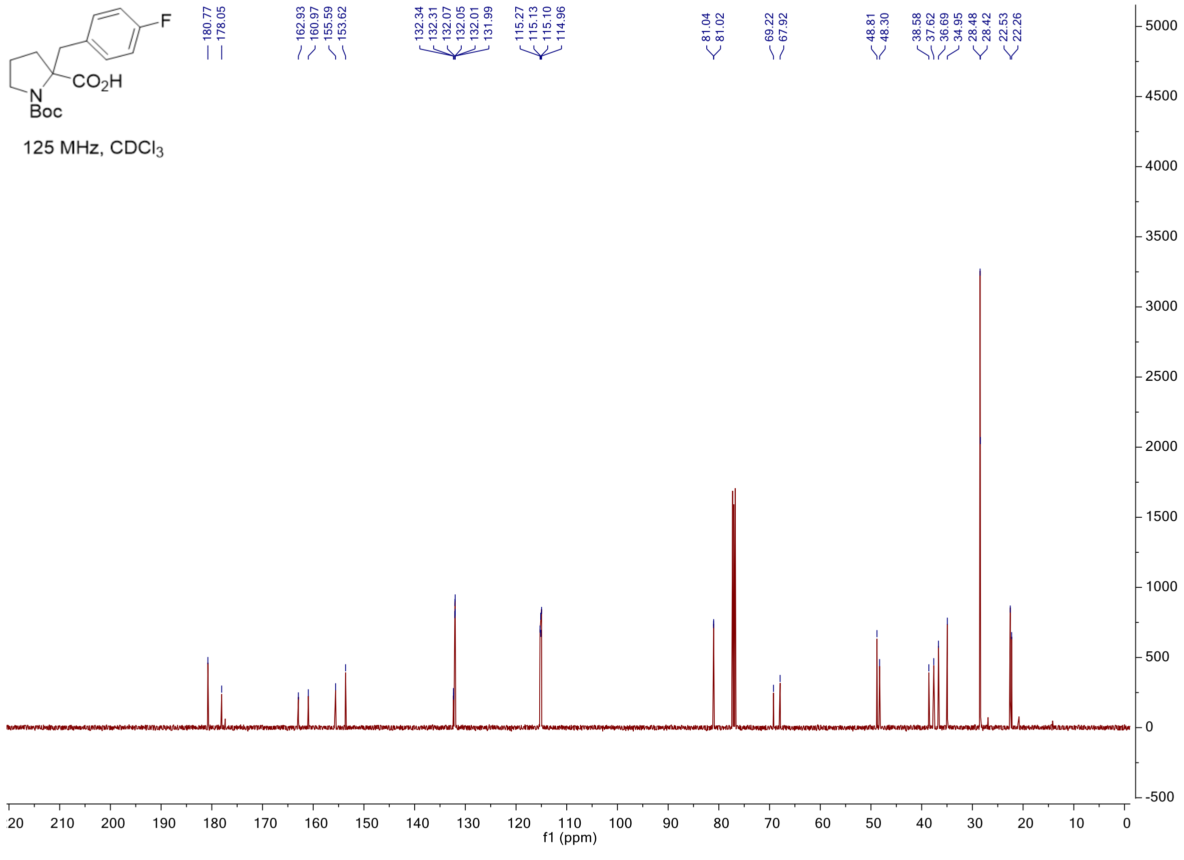

^{1}H and ^{13}C{^{1}H} Spectra of Compound **4e**:

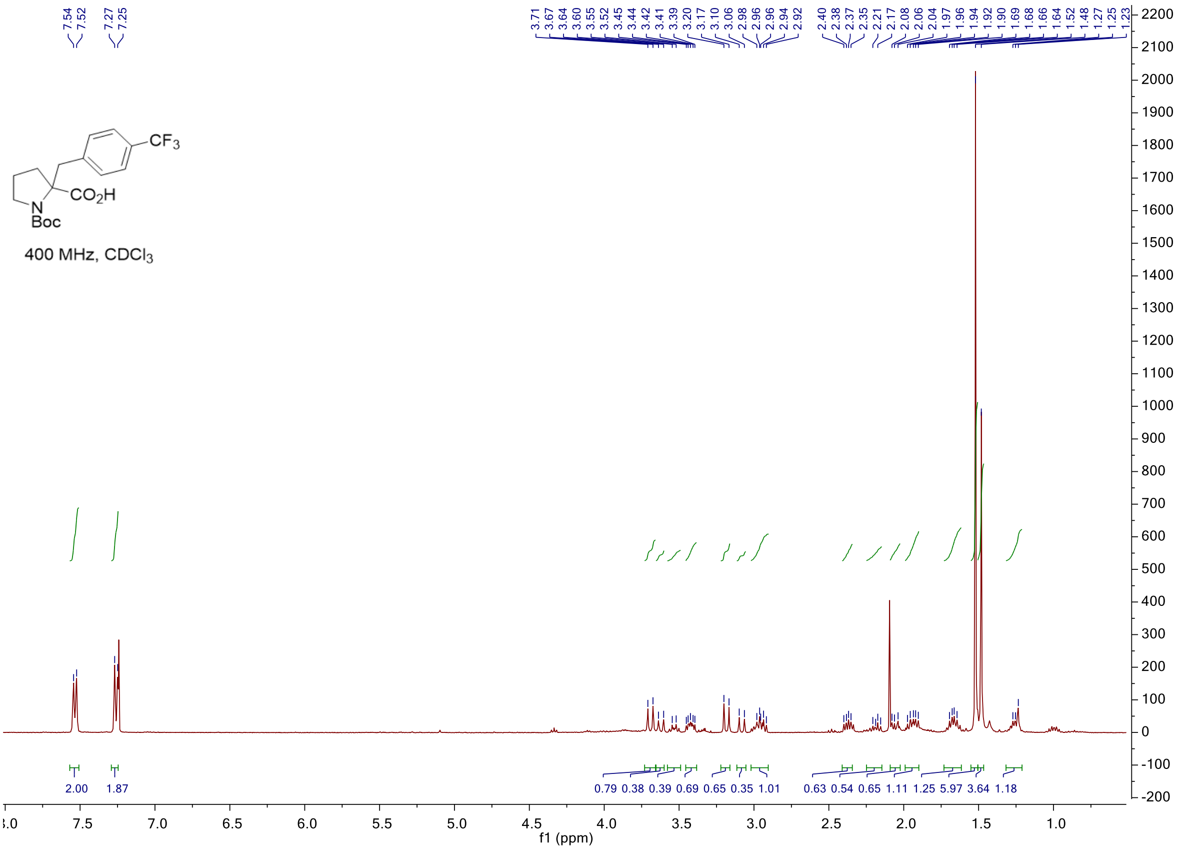

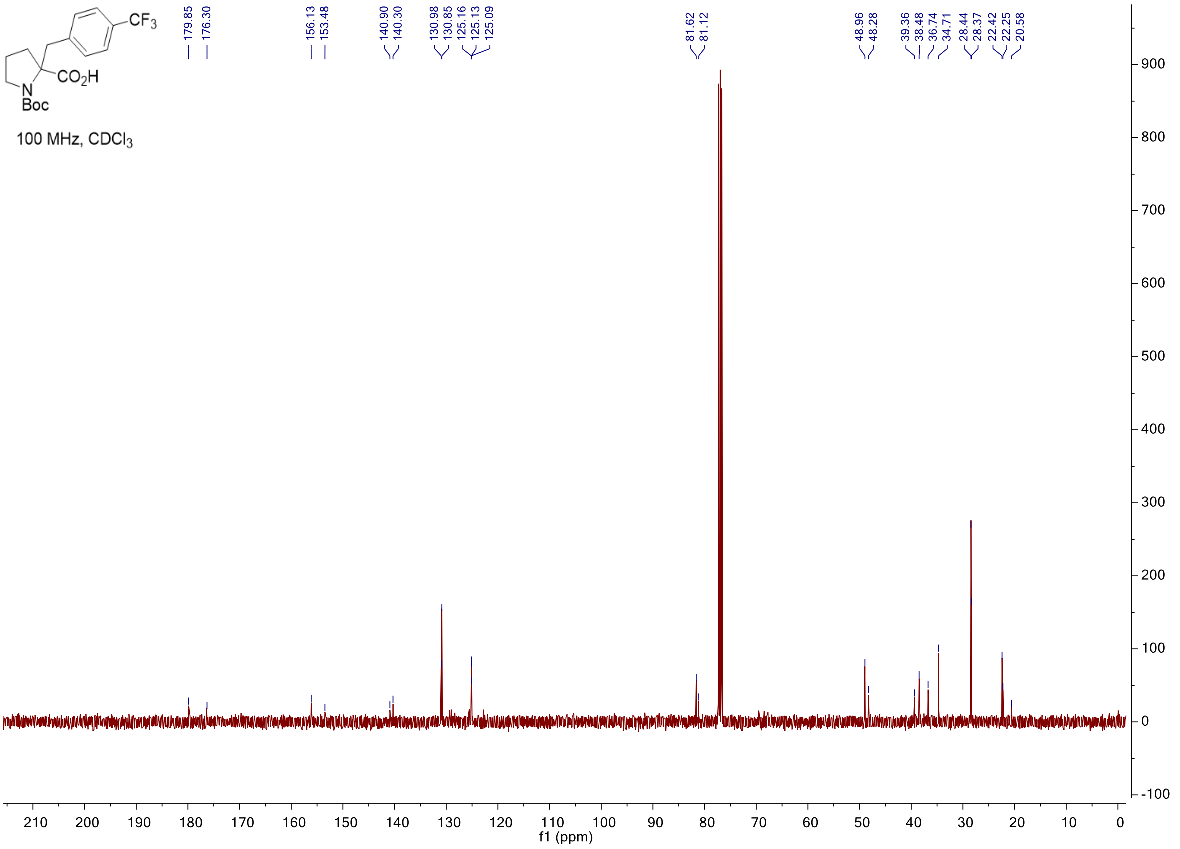

^{1}H and ^{13}C{^{1}H} Spectra of Compound **4f**:

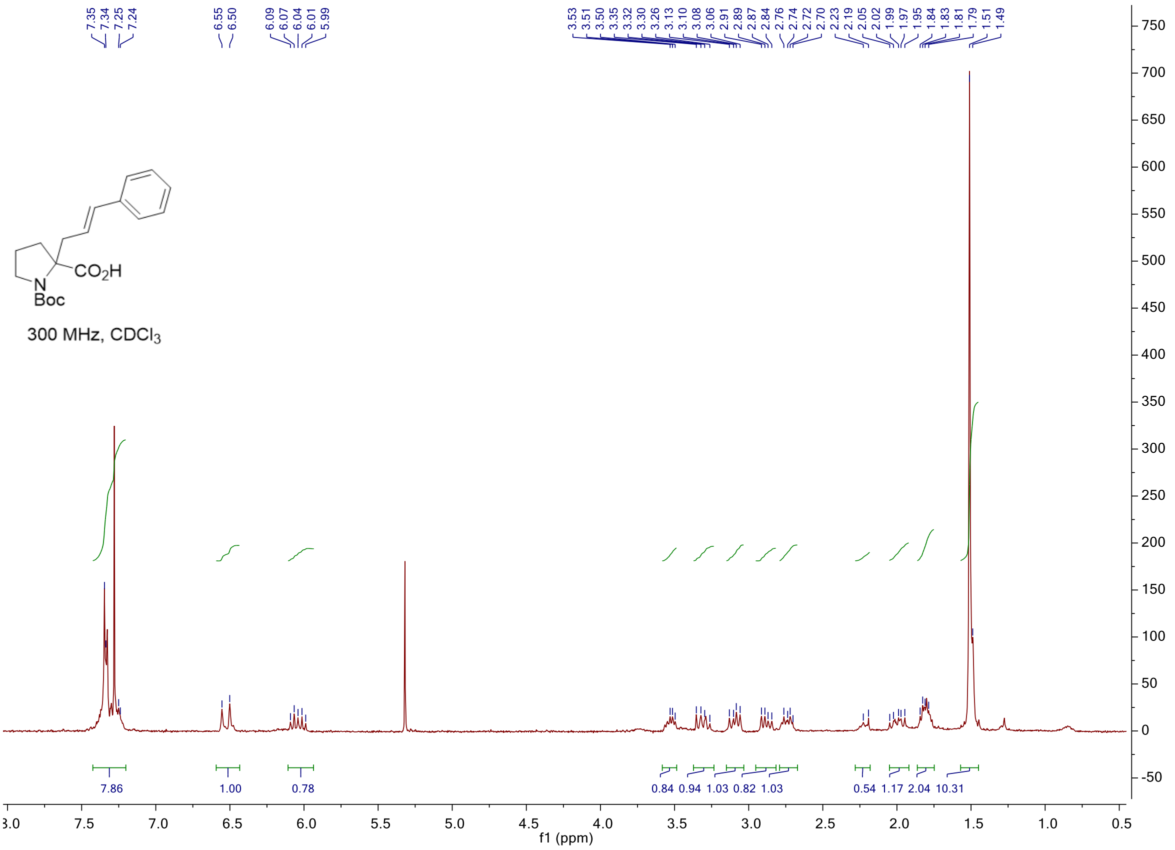

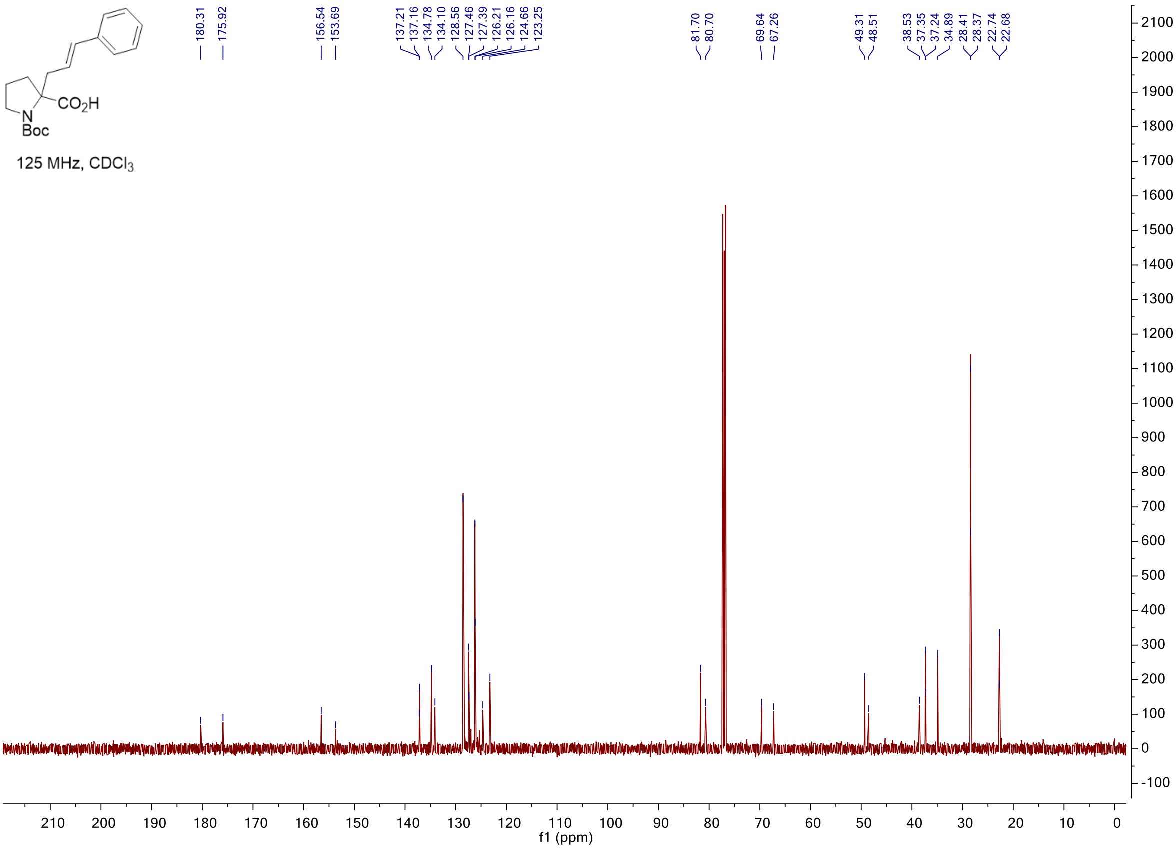

^{1}H and ^{13}C{^{1}H} Spectra of Compound **4g**:

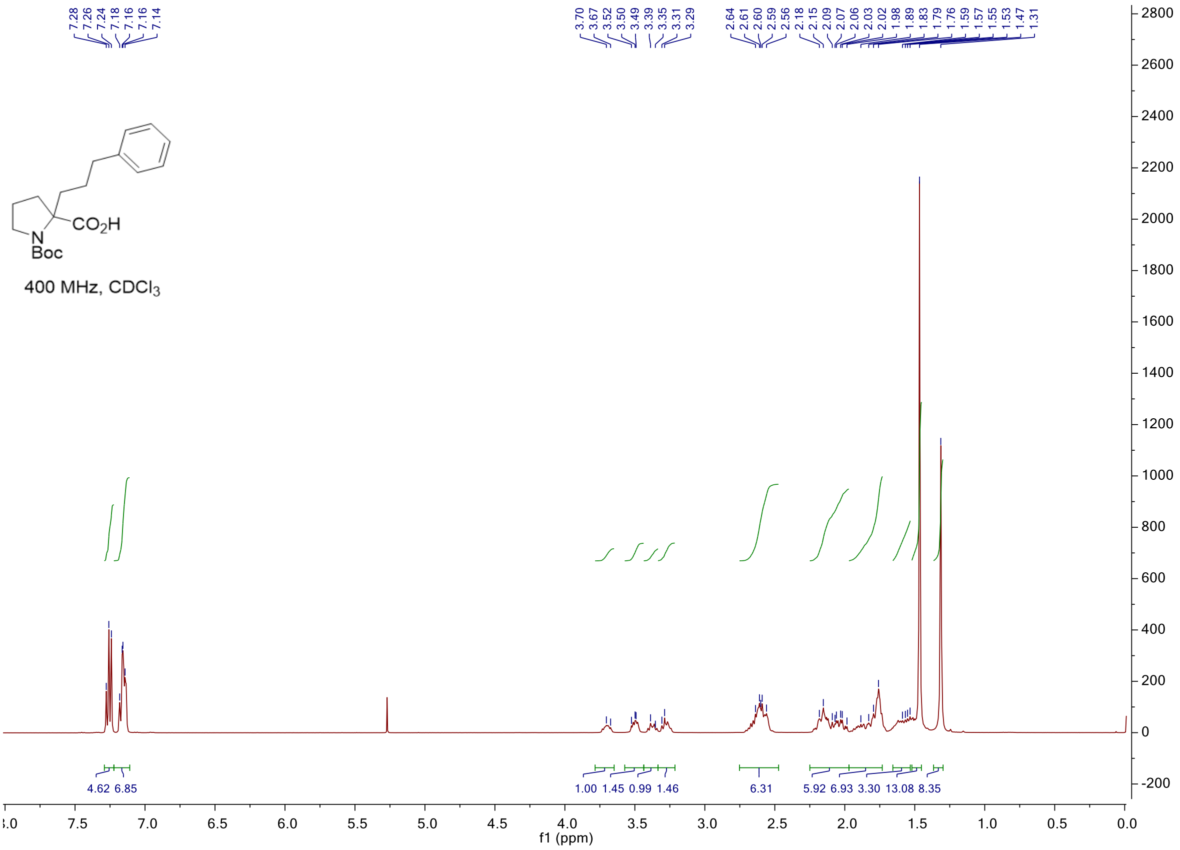

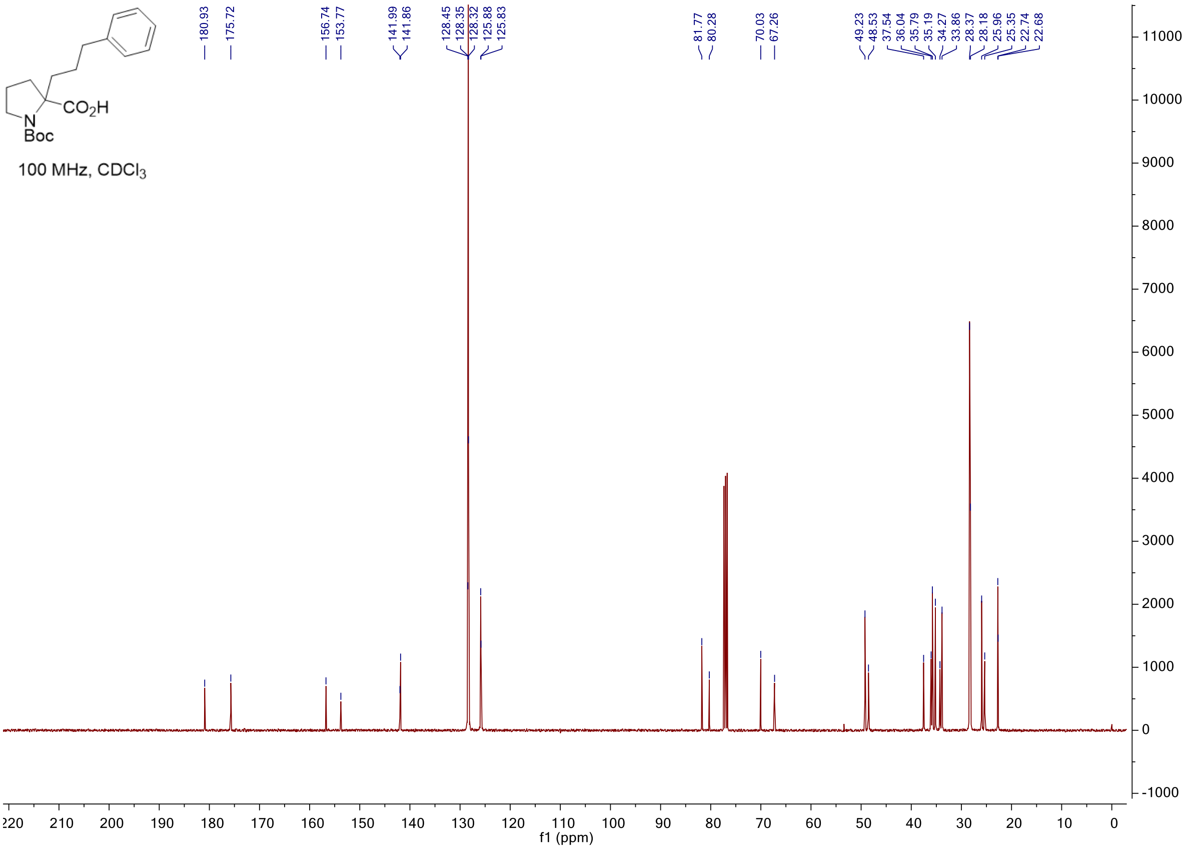

^{1}H and $^{13}C\{^{1}H\}$ Spectra of Compound **9a**:

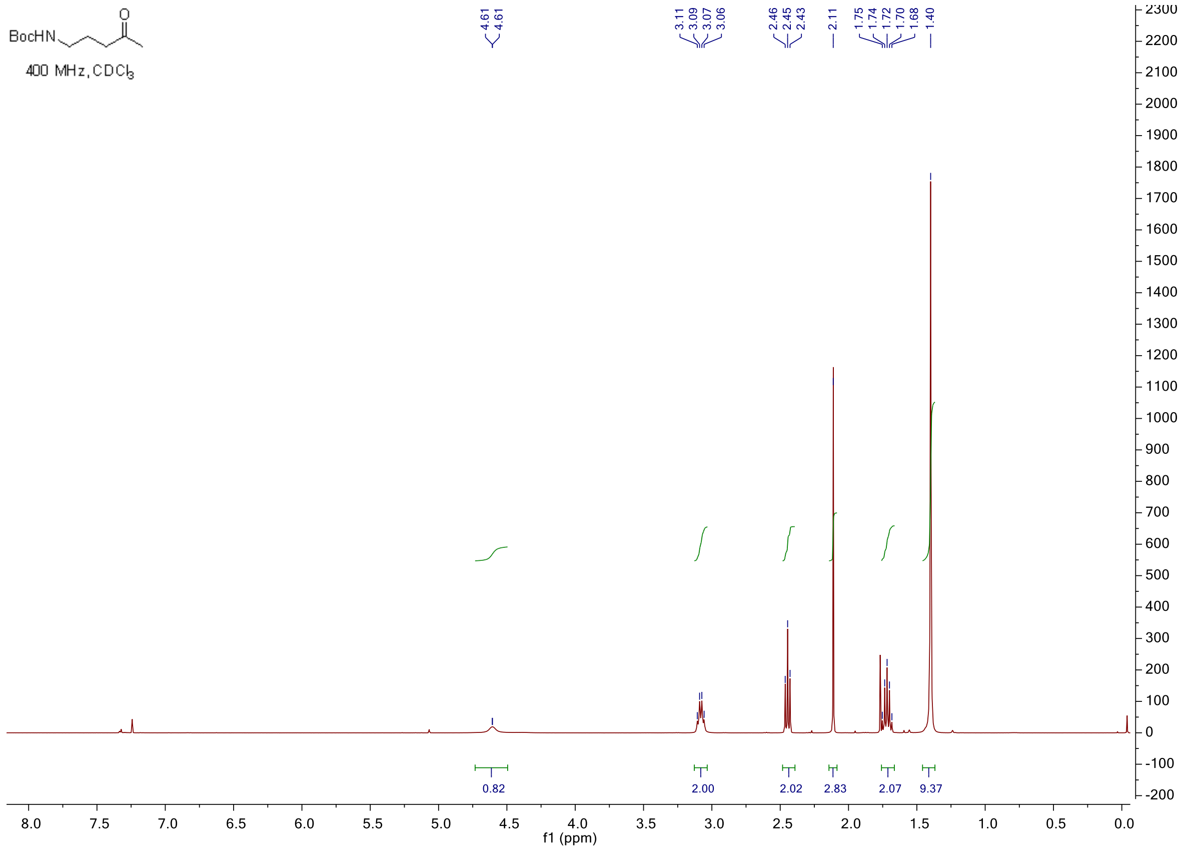

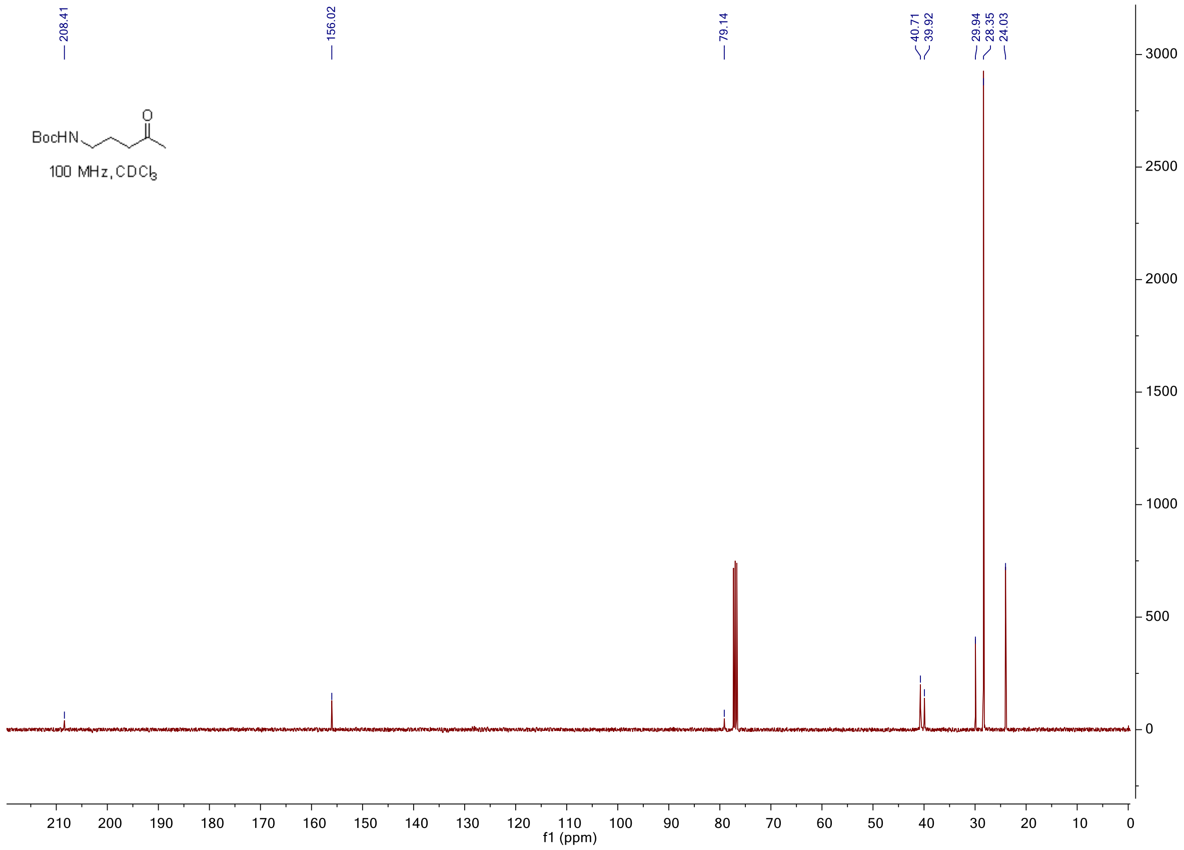

^{1}H and $^{13}C\{^{1}H\}$ Spectra of Compound **9b**:

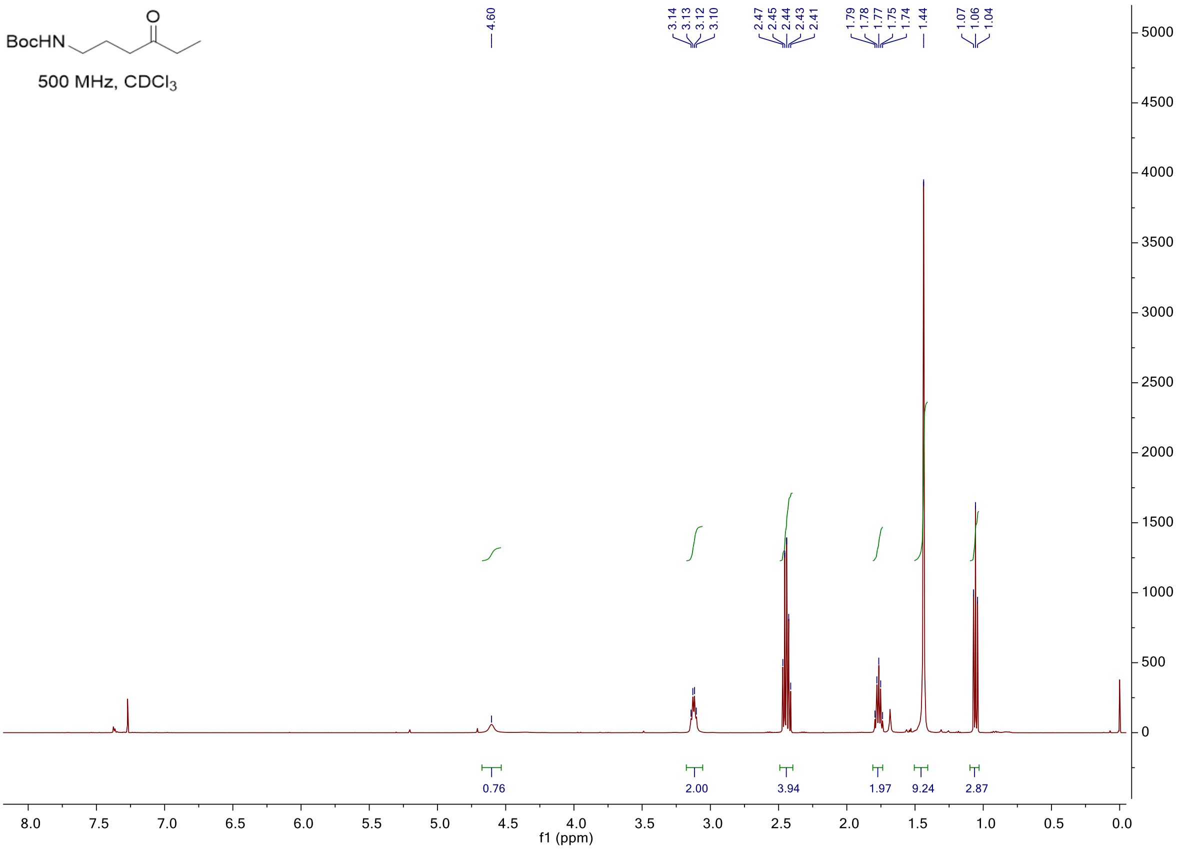

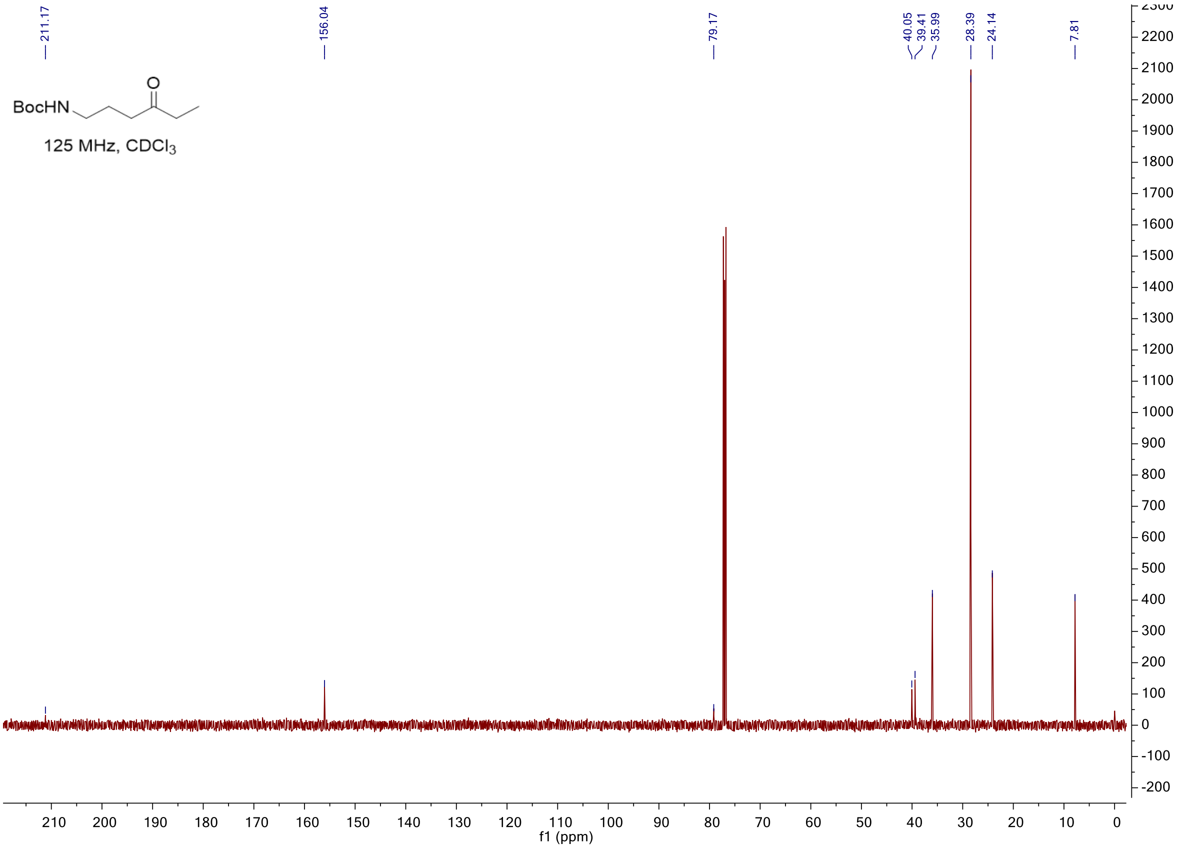

^{1}H and $^{13}C\{^{1}H\}$ Spectra of Compound **9c**:

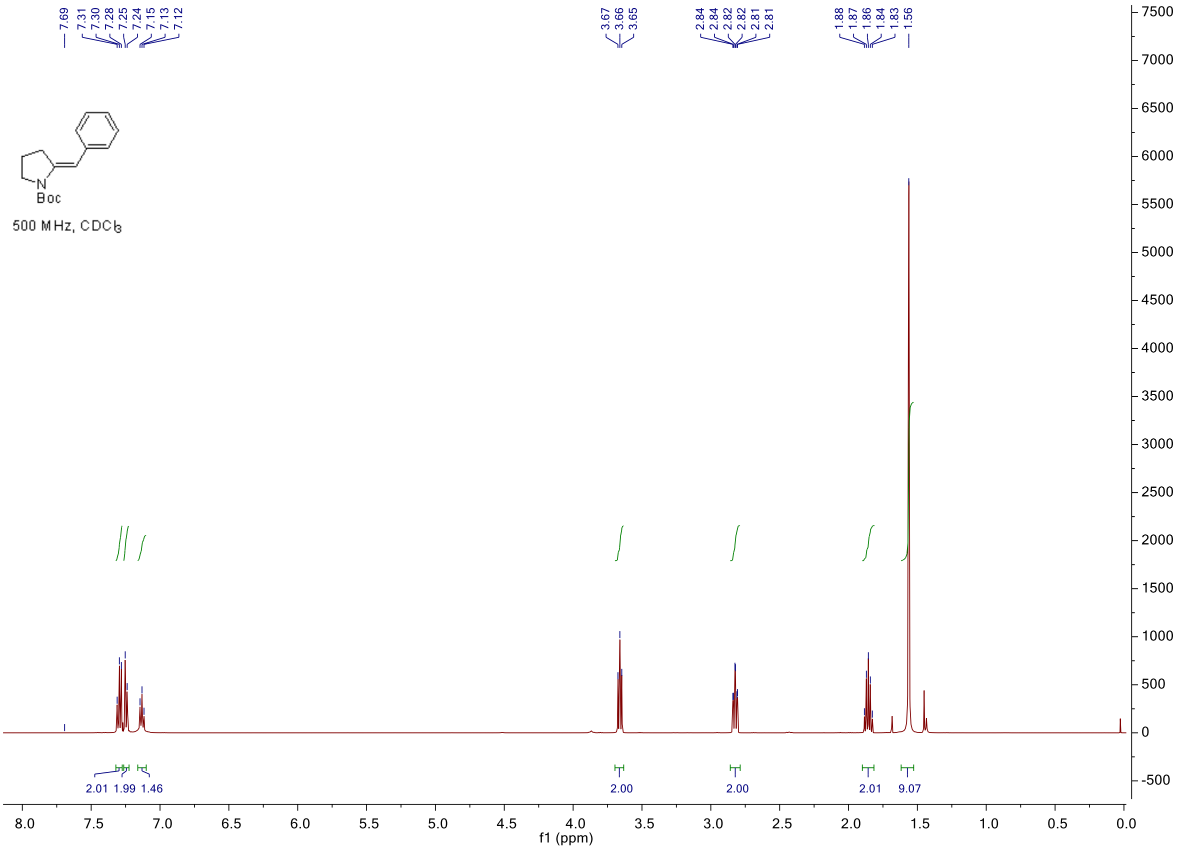

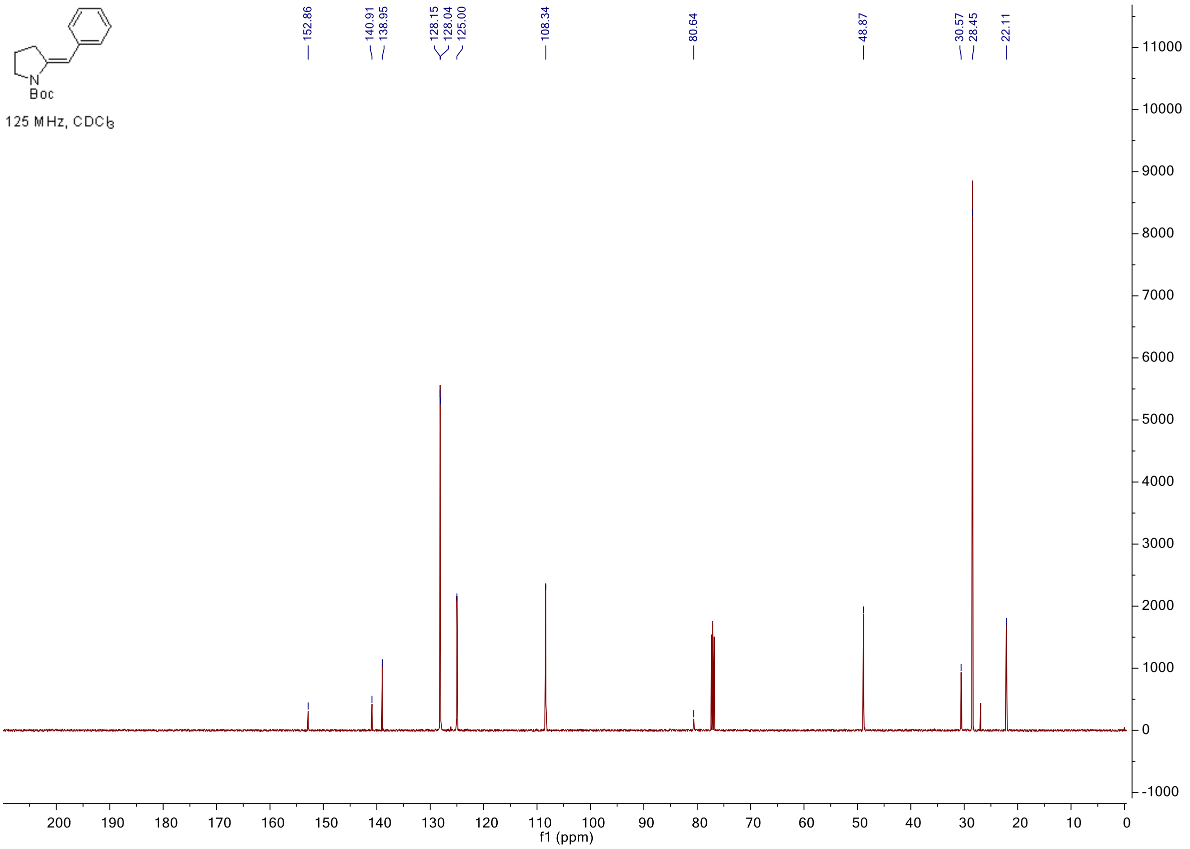

^{1}H, $^{13}C\{^{1}H\}$ and ^{19}F Spectra of Compound **9d**:

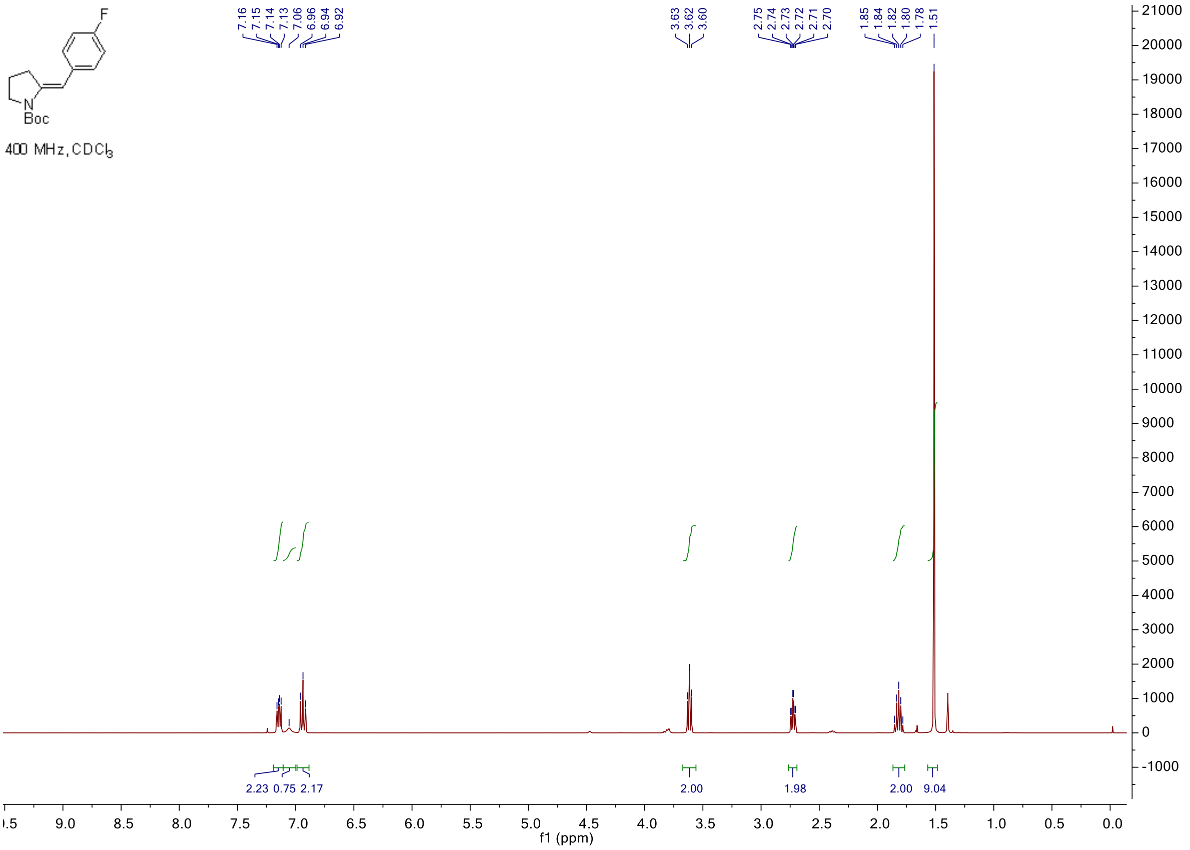

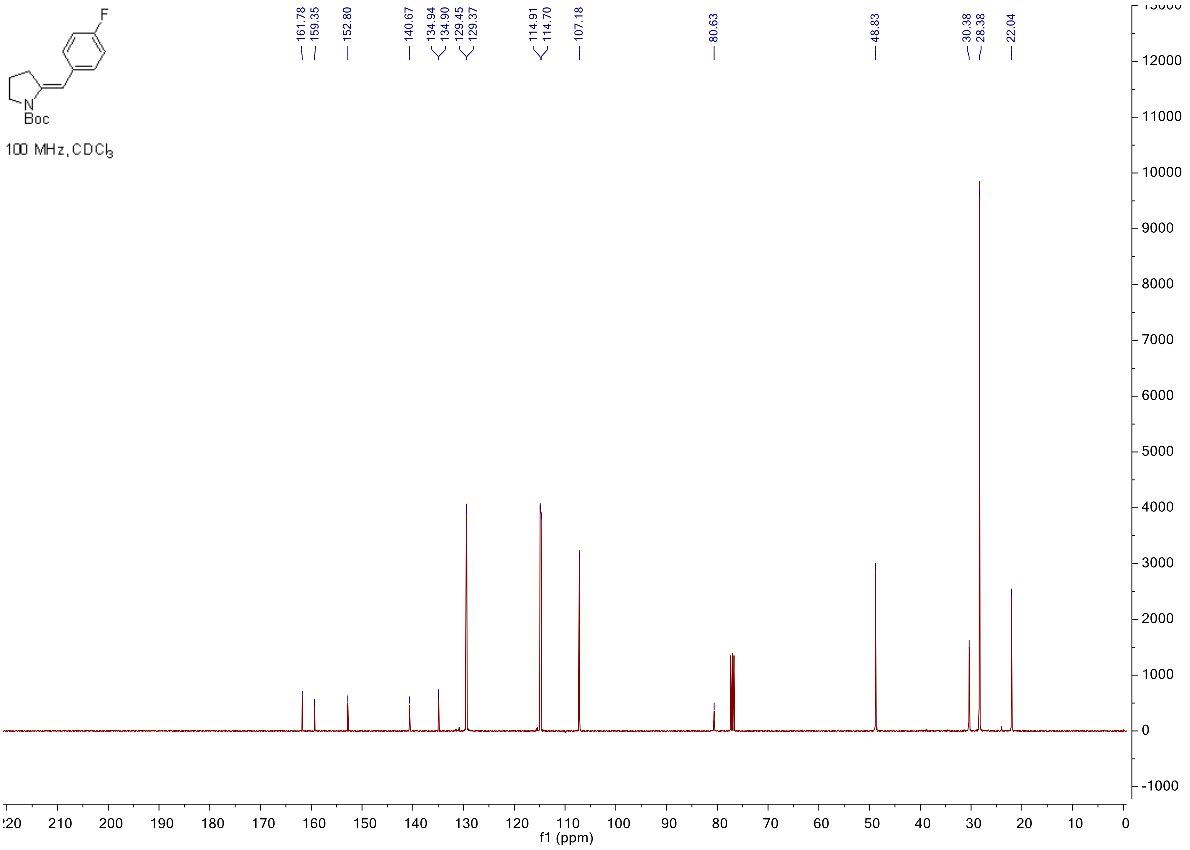

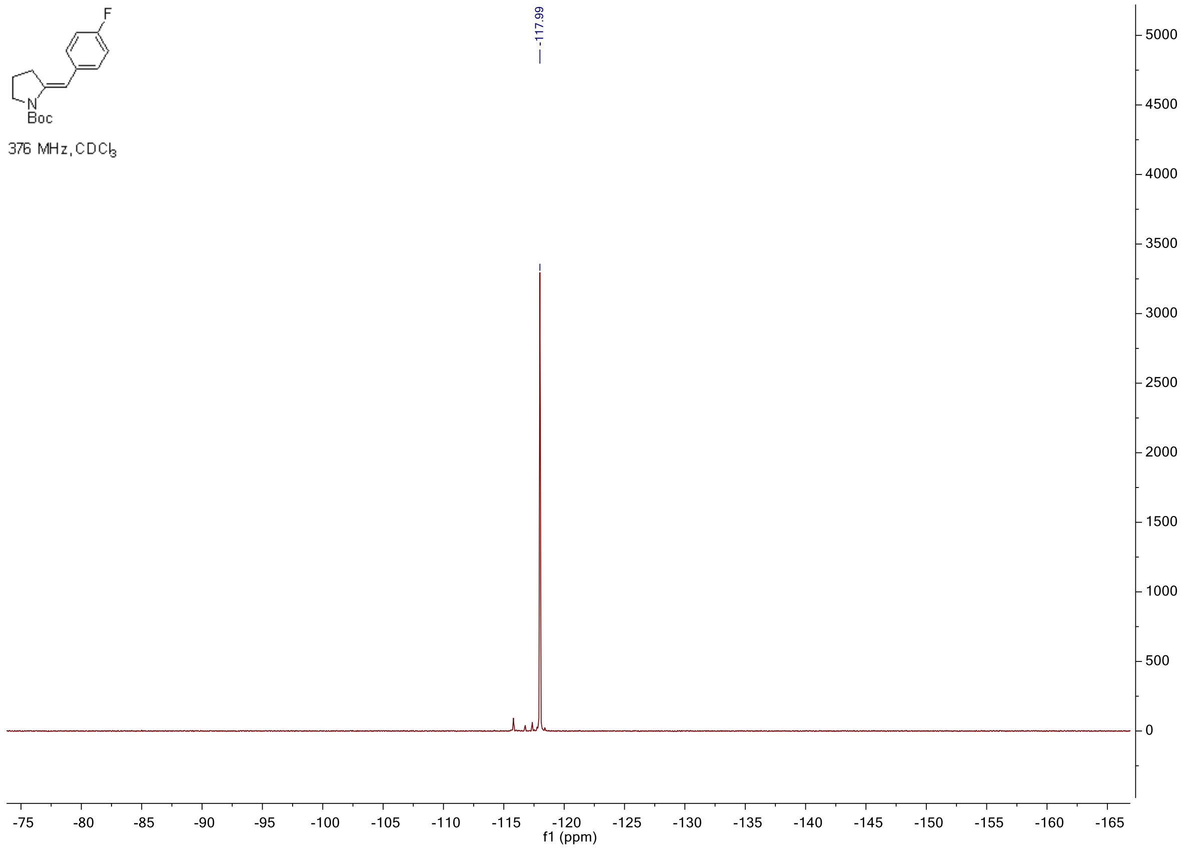

F
N
Boc
376 MHz, CDCl3
-117.99
f1 (ppm)

^{1}H, ^{13}C{^{1}H} and [19]F Spectra of Compound **9e**:

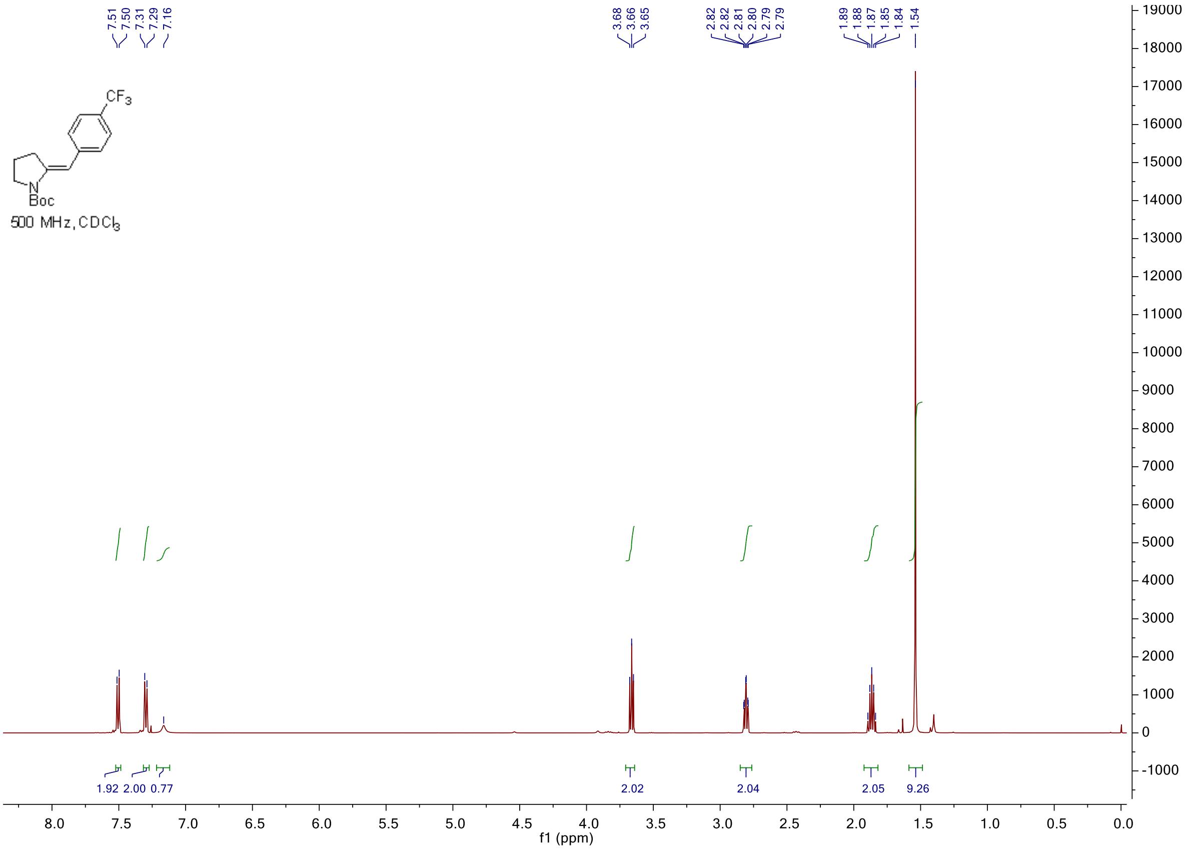

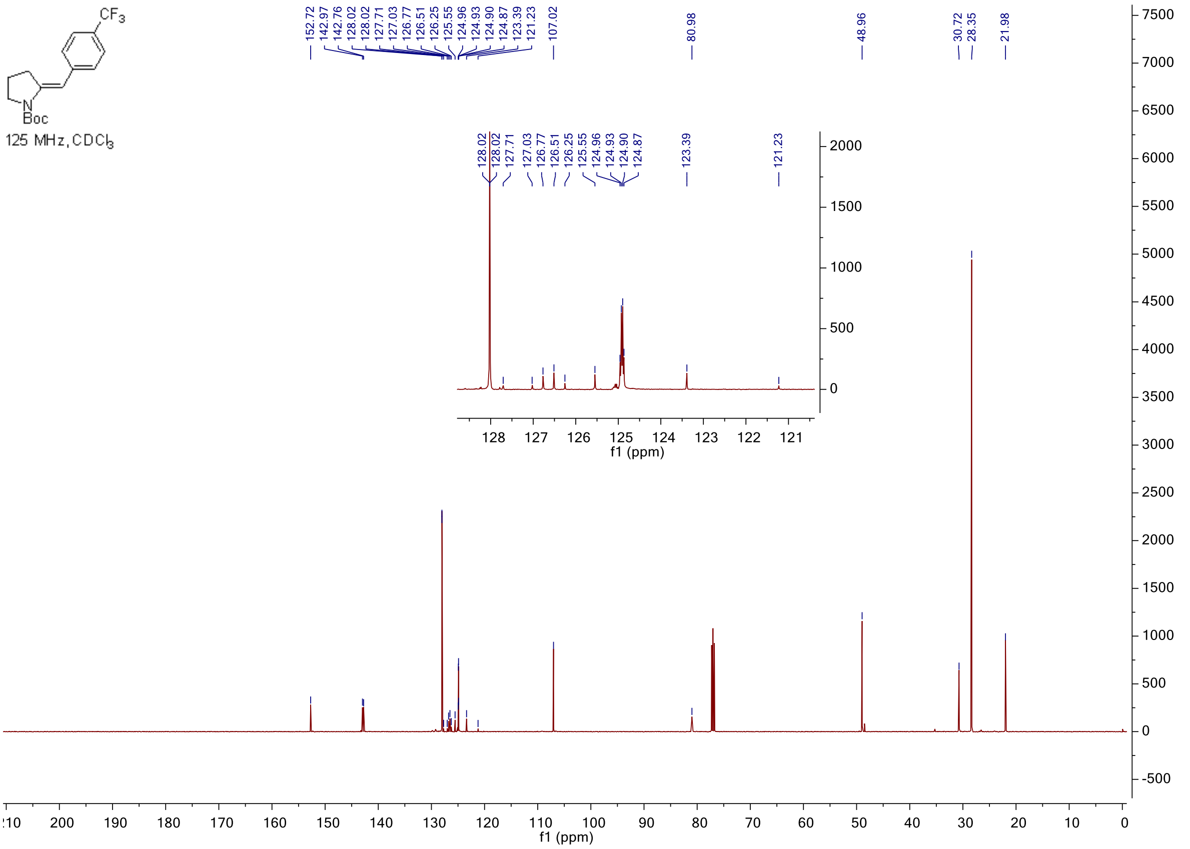

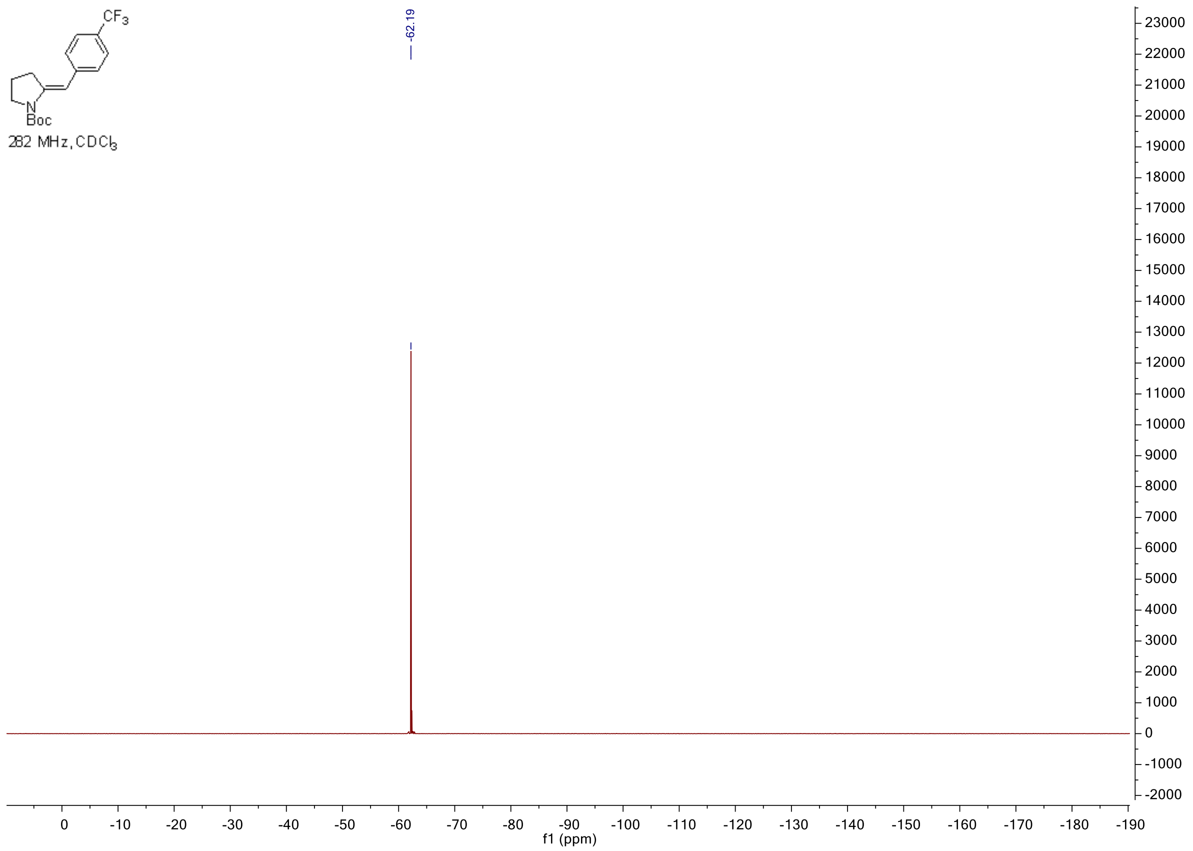

CF3
N
Boc
282 MHz, CDCl3
-62.19
f1 (ppm)

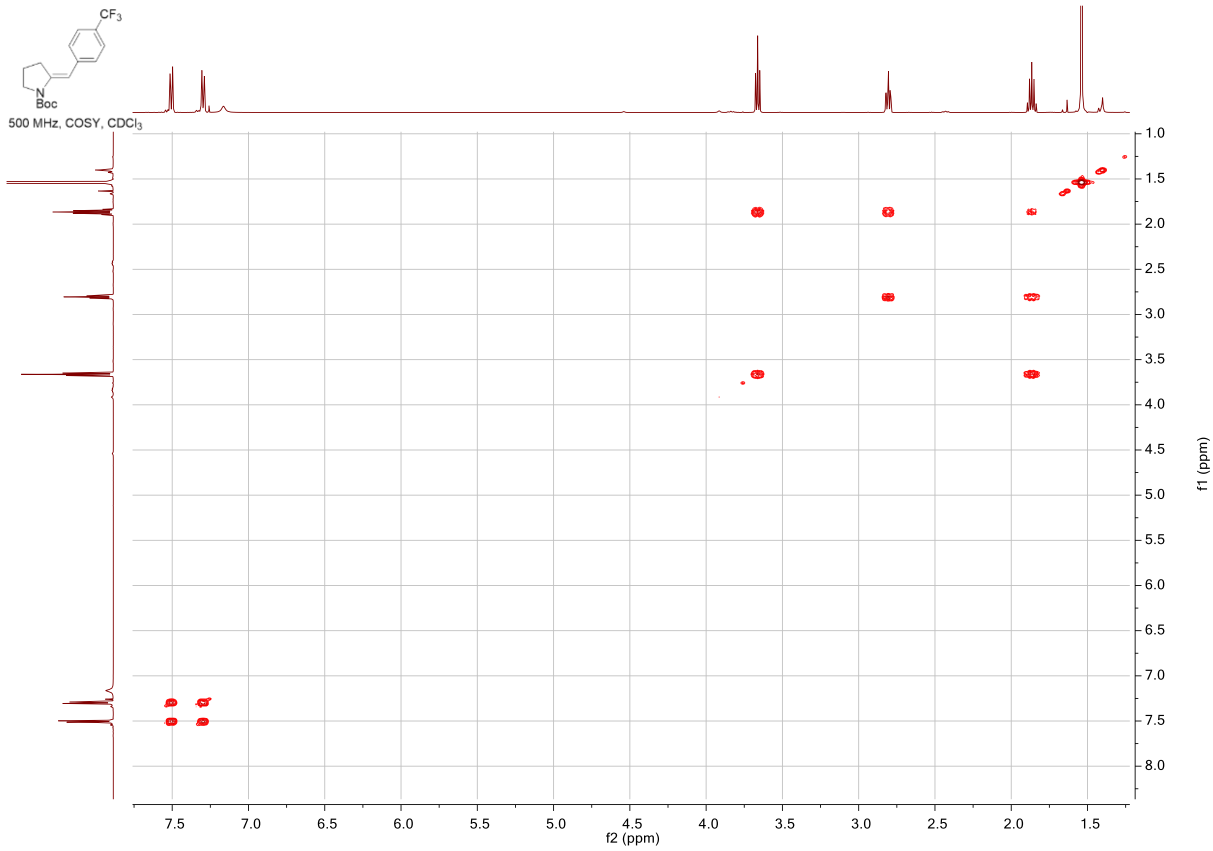

CF3
N
Boc
500 MHz, COSY, CDCl3
f2 (ppm)
f1 (ppm)

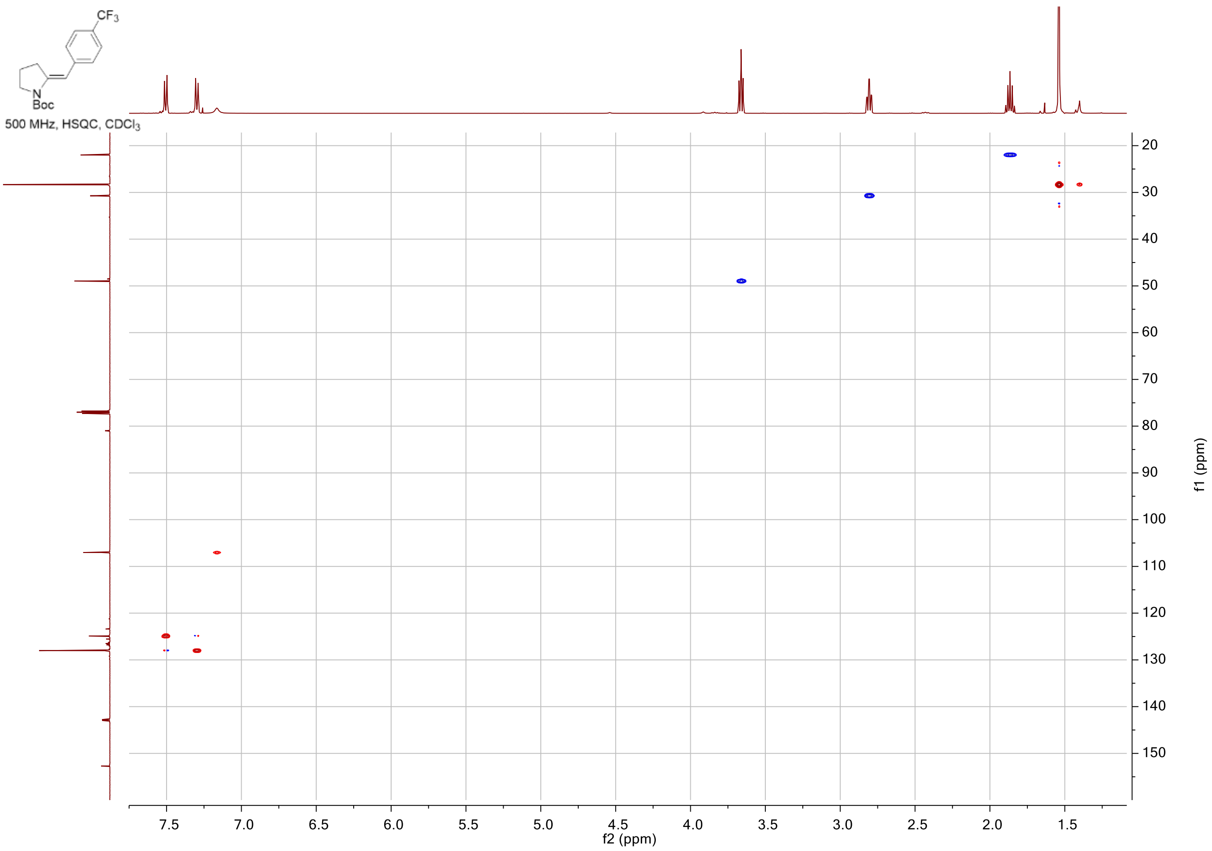
CF3
N
Boc
500 MHz, HSQC, CDCl3
f2 (ppm)
f1 (ppm)

^{1}H and ^{13}C{^{1}H} Spectra of Compound **9g**:

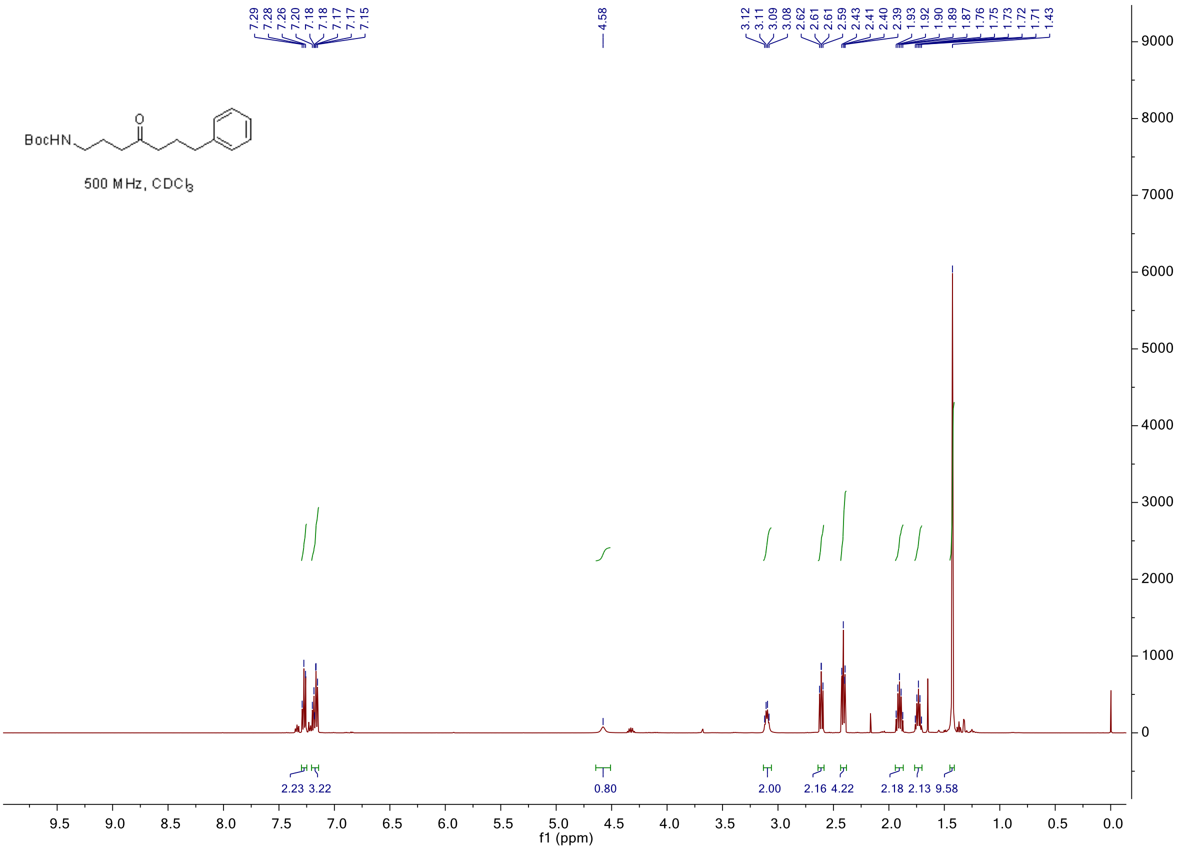

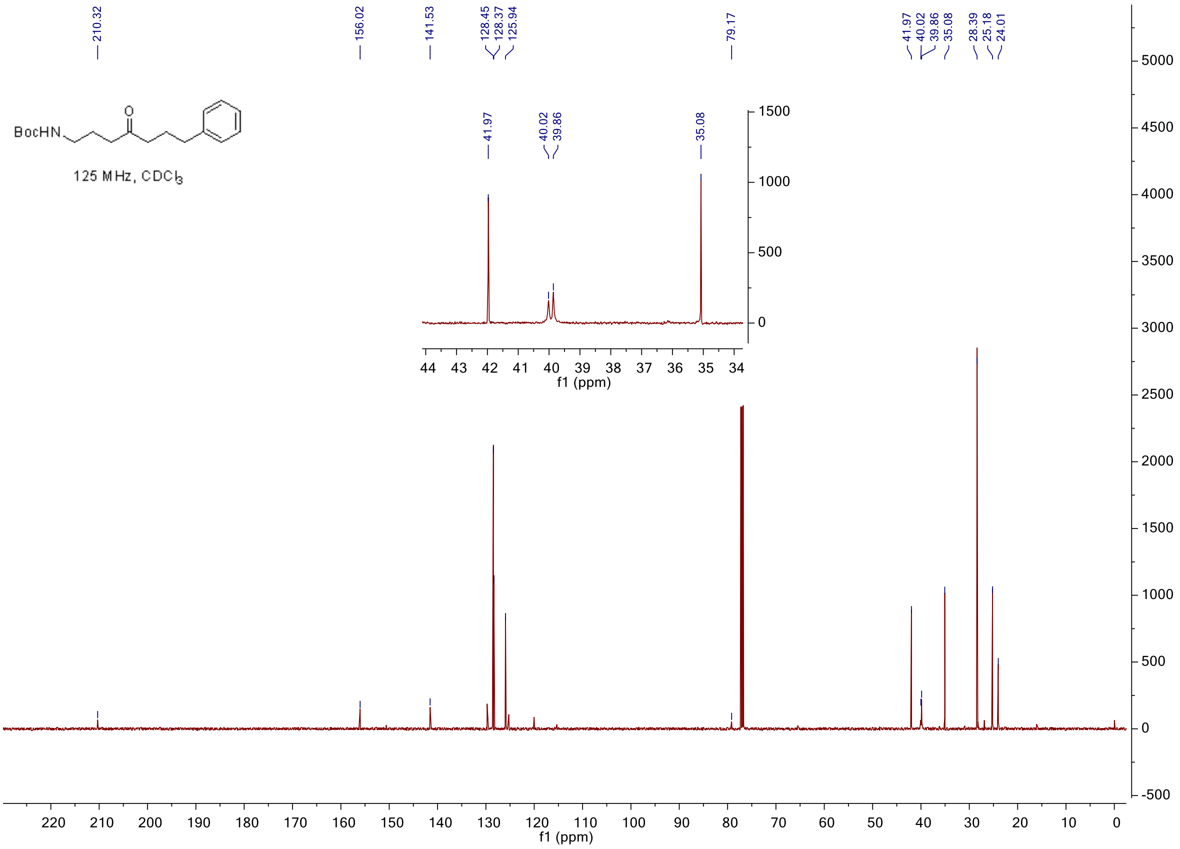

Supplement: Supplementary file 1 — jo1c01133_si_001.pdf [file jo1c01133_si_001.pdf]
